# Ordinal Characterization of Similarity Judgments

**Published:** 2023-10-11

**Authors:** Jonathan D. Victor, Guillermo Aguilar, Suniyya A. Waraich

**Affiliations:** Feil Family Brain and Mind Research Institute, Weill Cornell Medical College, 1300 York Avenue, New York, NY 10065

**Keywords:** perceptual spaces, maximum-likelihood estimation, ultrametric space, additive trees, triads, multidimensional scaling

## Abstract

Characterizing judgments of similarity within a perceptual or semantic domain, and making inferences about the underlying structure of this domain from these judgments, has an increasingly important role in cognitive and systems neuroscience. We present a new framework for this purpose that makes very limited assumptions about how perceptual distances are converted into similarity judgments. The approach starts from a dataset of empirical judgments of relative similarities: the fraction of times that a subject chooses one of two comparison stimuli to be more similar to a reference stimulus. These empirical judgments provide Bayesian estimates of underling choice probabilities. From these estimates, we derive three indices that characterize the set of judgments, measuring consistency with a symmetric dis-similarity, consistency with an ultrametric space, and consistency with an additive tree. We illustrate this approach with example psychophysical datasets of dis-similarity judgments in several visual domains and provide code that implements the analyses.

## Introduction

Characterization of the similarities between elements of a domain of sensory or semantic information is important for many reasons. First, these similarities, and the relationships between them, (Edelman, 1998; Kemp & Tenenbaum, 2008; Tversky, 1977), reveal the cognitive structure of the domain. Similarities are functionally important as they are the substrate for learning, generalization, and categorization (Kemp & Tenenbaum, 2008; Saxe, McClelland, & Ganguli, 2019; Shepard, 1958; Zaidi et al., 2013). At a mechanistic level, the quantification of similarities provides a way to test hypotheses concerning their neural substrates (Kriegeskorte & Kievit, 2013). Thus, measuring of perceptual similarities, and using these judgments to make inferences about the geometry of the underlying perceptual spaces, plays an important role in cognitive and systems neuroscience.

The goal of this work is to present a novel approach that complements the standard strategies used for this purpose. The starting point for the present approach, in common with standard strategies, is a set of triadic similarity judgments: is stimulus x or stimulus y more similar to a reference stimulus ? To make geometric inferences from such data, one standard approach is to make use of a variant of multidimensional scaling (de Leeuw & Heiser, 1982; Knoblauch & Maloney, 2008; Maloney & Yang, 2003; Tsogo, Masson, & Bardot, 2000; J. D. Victor, Rizvi, & Conte, 2017; Waraich & Victor, 2022), i.e., to associate the stimuli with points in a space, so that the distances between the points account for the perceptual similarities. Once these points are determined, inferences can be made about the dimensionality of the space, its curvature, and its topology. A second approach, topological data analysis, makes use of the distances directly, and then invokes graph-theoretic procedures (Dabaghian, Memoli, Frank, & Carlsson, 2012; Giusti, Pastalkova, Curto, & Itskov, 2015; Guidolin, Desroches, Victor, Purpura, & Rodrigues, 2022; Singh et al., 2008; Zhou, Smith, & Sharpee, 2018) to infer these geometric features.

In applying these approaches to experimental data, one must deal with the fact that even if a forced-choice response is required, the response likely represents an underlying choice probability – and that this choice probability may depend on sensory noise, noise in how distances are mentally computed and transformed into dis-similarities, and noise in the decision process in which dis-similarities are compared. As a consequence, analysis of an experimental dataset requires, at least implicitly, substantial assumptions. Such assumptions are not always benign: a monotonic transformation of distances – which preserves binary similarity judgments – can alter the dimensionality and curvature of a multidimensional scaling model (Kruskal & Wish, 1978). Topological data analysis via persistent homologies, which only makes use of rank orders of distances, is invariant to a global monotonic transformation of distances, but makes other assumptions (for example, that this transformation is the same across the domain), and does not typically take into account a noise model.

With these considerations in mind, here we pursue an approach to make inferences from the choice probabilities themselves, as estimated from repeated triadic judgments. Our main assumption is that if, for any particular triad, stimulus x is chosen more often than stimulus y as closer to a reference r, then the difference between x and r is less than the distance between y and r. Note that we do not make any assumptions about how relative or absolute distances are transformed into choice probabilities within an individual triad, or whether this transformation is the same across the domain.

While the limited nature of these assumptions necessary limits the inferences that can be made, the approach nevertheless can characterize a set of similarity judgments in three important ways. First, we ask whether the similarity judgments satisfy a relationship that is required for a symmetric notion of distance. Then, assuming that distances are symmetric, we derive an index of whether the judgments are consistent with an ultrametric space (Semmes, 2007), i.e., a set of distances that derives from a hierarchical representation. Finally, we index the extent to which the judgments are consistent with an additive tree, (Sattath & Tversky, 1977), a generalization of an ultrametric space. Each of these indices are graded, and can be viewed as quantifying the global characteristics of a set of similarity judgments, without the need to model how choice probabilities, distances, noise, and decision processes are related.

We illustrate the approach with several sample psychophysical datasets, and provide code to carry out these computations.

## Theory

### Overview and Preliminaries

Our goal is to develop indices that characterize a dataset of triadic similarity judgments, in a way that provides insight into the structure of the underlying perceptual space. Our central assumption is that, within a triadic judgment, the probability that a participant chooses one pair of stimuli as more similar than an alternative is monotonically related to the similarity. Typical datasets include large numbers of similarity judgments of overlapping triads, and the relationship between these judgments contains information about the underlying perceptual space. We show how this information can be accessed, without further making assumptions about the specifics of the monotonic relationship between choice probability and (dis)-similarity, whether it is constant throughout the space, or the decision process itself.

We focus on paradigms in which the basic unit of data collection and analysis is a triad (r;x,y), consisting of a stimulus r, designated as the reference, and two other stimuli, x and y designated as the comparison stimuli. The participant is asked to decide, in a forced-choice response, which of the two comparison stimuli is more similar to the reference. We consider this to be a probabilistic judgment, and denote the underlying probability that a participant judges x as more similar to r than y is to r as R(r;x,y).

The underlying probability R(r;x,y) is unknown, and must be estimated from the trials in which the triad (r;x,y) is presented. We denote the number of such trials by N(r;x,y) and use C(r;x,y) to denote the number of trials in which the participant judges x as more similar to r than y is to r. These provide a naïve estimate $R_{obs}(r;x,y)$ of the choice probability:

(1.1) $$R_{\text{obs}}\left(r;x,y\right)=\frac{C\left(r;x,y\right)}{N\left(r;x,y\right)}.$$

We assume that the experimental procedure guarantees that the two comparison stimuli of a triad are treated identically. (One way to guarantee this is to randomly swap or balance the positions of x and y across trials.) With this assumption,

(1.2) $$\begin{array}{c} C(r;x,y)+C(r;y,x)=N(r;x,y)=N(r;y,x), \end{array}$$

(1.3) $$R_{\text{obs}}(r;x,y)+R_{\text{obs}}(r;y,x)=1,$$

and

(1.4) R(r;x,y)+R(r;y,x)=1.

A triadic judgment is the result of a two-step process: first, estimation of the dis-similarity between the reference and each of the comparison stimuli, and second, comparison of these dis-similarities. We denote the dis-similarity of a comparison stimulus z to a reference stimulus r by D(r,z). Our central assumption is that a participant is more likely to judge that x is more similar to r than y is to r if, and only if, D(r,x)<D(r,y). That is,

(1.5) R(r;x,y)>1/2⇔D(r,x)<D(r,y).

An immediate consequence is that a choice probability of exactly 1/2 only occurs when dis-similarities are exactly equal: combining (1.4) with (1.5) yields

(1.6) R(r;x,y)=1/2⇔D(r,x)=D(r,y),

We also assume that dis-similarities are non-negative, and that a dis-similarity of zero only occurs for stimuli that are identical:

(1.7) D(x,y)≥0 and D(x,y)=0⇔x=y.

The central assumption embodied by eq. (1.5)- that choice probabilities reflect rank order of dis-similarities – stops short of making a more quantitative assumption about the relationship between the choice probability and perceived dis-similarities. Specifically, we only make use of the sign of the comparison- for example, that an alligator and toothpaste are more dis-similar than an alligator and a panda. We do not attempt to infer the size of this difference, in absolute terms, relative to an internal noise, or relative to the dis-similarity of another pair of stimuli that was not explicitly compared within a triad.

The present analysis, which applies most directly to a paradigm in which each trial is devoted to judgment of a single triad, is applicable to other paradigms in which individual trials yield judgments about more than one triad, provided that each judgment can be considered to be independent of the context in which it is made. For example, in the “odd one out” paradigm (also known as the “oddity task” (Kingdom & Prins, 2016)), three stimuli are presented and the participant is asked to choose which one is the outlier. Here, a selection of a stimulus $x_{j}$ out of a triplet $\left\{x_{j},x_{k},x_{l}\right\}$ can be interpreted as a judgment that $D\left(x_{k},x_{j}\right)>D\left(x_{k},x_{l}\right)$ and also that $D\left(x_{l},x_{j}\right)>D\left(x_{l},x_{k}\right)$, and thus contributes to estimates of choice probabilities for two triads, $\left(x_{k};x_{j},x_{l}\right)$ and $\left(x_{l};x_{j},x_{k}\right)$. The analysis is also applicable to paradigms in which the participant is asked to rank m comparison stimuli $x_{1},\ldots ,x_{m}$ in order of similarity to a reference stimulus r (Waraich & Victor, 2022). The ranking obtained on each trial then contributes to estimation of choice probabilities for $\left(\begin{array}{l} m\\ 2 \end{array}\right)$ triads $\left(r;x_{k},x_{l}\right)$, one for each pair of comparison stimuli.

We do not require that the experiment explore all triads (in an experiment with M stimuli, there are $M\left(\begin{array}{c} M-1\\ 2 \end{array}\right)=\frac{1}{2}M(M-1)(M-2)$ distinct triads ), but the incisiveness of the approach will naturally improve as more triads are explored and as each triad is presented more often, so that $R_{obs}(r;x,y)$ is a better estimator of R(r;x,y).

### Assessing symmetry

The first index we develop tests the extent to which the dis-similarities underlying the perceptual judgments are consistent with a symmetric distance, i.e., a distance in which the reference and comparison stimuli are treated alike. To do this, we first identify necessary conditions on the dis-similarities D(x,y) required by this symmetry. This yields a set of inequalities that the underlying choice probabilities R(r;x,y) must satisfy. Then, we take a Bayesian approach: given the observed data $R_{obs}(r;x,y)$, which are only estimates of the choice probabilities R(r;x,y), we determine the likelihood that these choice probabilities R(r;x,y) are consistent with the requisite inequalities.

#### (2.3) A necessary condition for symmetry

To derive conditions on the choice probabilities that are necessary for symmetric dis-similarities, we first note that if the dis-similarity is symmetric, then at least one of the three inequalities

(2.1) $$\begin{array}{r} D\left(x,y\right)<D\left(x,z\right)\\ D\left(z,x\right)<D\left(z,y\right)\\ D\left(y,z\right)<D\left(y,x\right) \end{array}\}$$

must be false. For if all of the inequalities (2.1) held, and the dis-similarity is symmetric, alternate application of one of (2.1) and the assumption of symmetry would lead to a contradiction

(2.2) D(x,y)<D(x,z)=D(z,x)<D(z,y)=D(y,z)<D(y,x),

as a quantity cannot be less than itself.

To translate the condition that at least one of (2.1) must be false into a condition on the choice probabilities, we use the central hypothesis, that dis-similarities and choice probabilities are monotonically related within a triad (eq. (1.5)). If at least one of (2.1) must be false, then at least one of the inequalities

(2.3) $$\begin{array}{r} R\left(x;y,z\right)>1/2\\ R\left(z;x,y\right)>1/2\\ R\left(y;z,x\right)>1/2 \end{array}\}$$

must consequently be false. Similarly (reversing all the inequalities in (2.1)), at one of the inequalities

(2.4) $$\begin{array}{r} R\left(x;y,z\right)<1/2\\ R\left(z;x,y\right)<1/2\\ R\left(y;z,x\right)<1/2 \end{array}\}$$

must be false as well. A slightly stronger statement is that at least one of (2.3) and at least one of (2.4) must be false, even when two of the three inequalities in either set are replaced by an inclusive inequality. (These borderline cases, while not crucial here, will be important for testing the ultrametric property below.)

These conditions can be summarized as follows: if at least one of the triplet of choice probabilities R(x;y,z),R(z;x,y), and R(y;z,x) is strictly greater than 1/2, then at least one of them must also be strictly less than 1/2, and vice-versa. Put another way: the triplet of choice probabilities R(x;y,z),R(z;x,y), and R(y;z,x) can only reflect a symmetric dis-similarity if the triplet of values lies in a specific subset $S_{sym}$ of [0,1]^3^. Other than boundary points, $S_{sym}$ consists of the cube [0,1]^3^ from which two smaller cubes, ${\left[\frac{1}{2},1\right]}^{3}$ (eq. (2.3)) and ${\left[0,\frac{1}{2}\right]}^{3}$ (eq. (2.4)), are removed.

#### Likelihood ratio calculation and an index

We now use the observed data to determine the likelihood that, given observed responses for a triplet of triads (x;y,z),(z;x,y), and (y;z,x), the corresponding triplet of choice probabilities lies in $S_{sym}$, vs. its complement $[0,1{]}^{3}\setminus S_{sym}$. We use a Bayesian approach: the a posteriori likelihood of a triplet of choice probabilities R(x;y,z),R(z;x,y), and R(y;z,x) is proportional to the product of their prior probabilities, and the probability that they will lead to the observed responses. We then integrate this product over the space of choice probabilities in which, for each triplet, at least one of (2.3) and at least one of (2.4) is false. This space is a product of the domains $S_{\text{sym}}$ for each triplet, which we denote by $\Omega_{sym}$. We compare this integral with an integral of the a posteriori likelihood over all possible choice probabilities, which we denote Ω, to assess how much of this total likelihood is consistent with symmetry. To carry out these integrations, we exploit the fact that the constraints that define $\Omega_{sym}$ are grouped into triplets, and the choice probabilities in each triplet are disjoint. Because of this disjointness, the integral over $\Omega_{sym}$ factors into a product of integrals over multiple copies of the domain $S_{sym}$, with one copy for each triplet.

To begin the Bayesian approach, we choose a product of Dirichlet distributions (Ferguson, 1973) of identical shape as the prior for the set of choice probabilities. This choice is both analytically convenient and practical for real data (see Results), but it is not essential to the approach. As each probability is univariate, the Dirichlet prior reduces to a beta function, so our prior is that each choice probability is independently distributed according to

(2.5) $$p_{a,b}(R)≜\frac{1}{B\left(a,b\right)}R^{a-1}(1-R{)}^{b-1}.$$

Here, B(a,b) is the beta-function, defined in the standard fashion by

(2.6) $$B\left(a,b\right)≜\overset{1}{\underset{0}{\int }}u^{a-1}{\left(1-u\right)}^{b-1}du=\frac{\Gamma \left(a\right)\Gamma \left(b\right)}{\Gamma \left(a+b\right)}.$$

Since R(r;x,y)=1-R(r;y,x), the prior must be symmetric, so we can take a=b in (2.5):

(2.7) $$p_{a}(R)≜\frac{1}{B(a,a)}R^{a-1}(1-R{)}^{a-1}\text{,}$$

We determine the Dirichlet parameter a by maximizing the likelihood of the observed set of choice probabilities, assuming that the individual responses for the i th triad (r;x,y) are independently drawn from a Bernoulli distribution with parameter $R_{i}=R(r;x,y)$. Given $R_{i}$, the probability that the subject reports D(r,x)<D(r,y) in $C_{i}$ of $N_{i}$ presentations is

(2.8) $$p\left(C_{i}|R_{i},N_{i}\right)=\left(\begin{array}{l} N_{i}\\ C_{i} \end{array}\right)R_{i}^{C_{i}}{\left(1-R_{i}\right)}^{N_{i}-C_{i}}.$$

Integrating over the prior (2.7) for $R_{i}$ yields the probability of observing $C_{i}$ reports of D(r,x)<D(r,y) in $N_{i}$ presentations, given the Dirichlet parameter :

(2.9) $$p\left(C_{i}|N_{i},a\right)=\left(\begin{array}{c} N_{i}\\ C_{i} \end{array}\right)\overset{1}{\underset{0}{\int }}\left(\frac{1}{B\left(a,a\right)}R_{i}^{a-1}{\left(1-R_{i}\right)}^{a-1}\right)R_{i}^{C_{i}}{\left(1-R_{i}\right)}^{N_{i}-C_{i}}dR_{i}\;=\left(\begin{array}{c} N_{i}\\ C_{i} \end{array}\right)\frac{B\left(a+C_{i},a+N_{i}-C_{i}\right)}{B\left(a,a\right)}.$$

Making use of the independence of each triad yields the overall log-likelihood:

(2.10) $$LL\left(a\right)=\text{log}\left(\underset{i}{\prod }p\left(C_{i}|N_{i},a\right)\right)=\text{log}K+\underset{i}{\sum }\text{log}\frac{B\left(a+C_{i},a+N_{i}-C_{i}\right)}{B\left(a,a\right)},$$

where K is a combinatorial factor independent of a, and the sum ranges over all triads. The value of a that maximizes (2.10), along with (2.7), defines the prior for the set of choice probabilities:

(2.11) $$P\left(\vec{R}\right)=\underset{i}{\prod }(\frac{1}{B\left(a,a\right)}R_{i}^{a-1}{\left(1-R_{i}\right)}^{a-1}),$$

where $\vec{R}$ denotes the set of choice probabilities for all triads.

Via Bayes rule, this prior determines the posterior likelihood of a set of choice probabilities:

(2.12) $$p\left(\vec{R}|\vec{C},\vec{N}\right)=\frac{p\left(\vec{C}|\vec{R},\vec{N}\right)P\left(\vec{R}\right)}{p(\vec{C},\vec{N}).}$$

where $\vec{C}$ denotes the responses $C_{i}$ to each of the triads, $\vec{N}$ denotes the number of times that each was presented, and $\vec{R}$ denotes the underlying choice probabilities.

The key step is to compare the likelihood that the observations $\vec{C}$ result from underlying choice probabilities within the subset $\Omega_{sym}$ in which the requisite inequalities hold, to the likelihood that the observations result from choice probabilities within the entire space of choice probabilities, Ω. We denote these quantities by

(2.13) $$L_{\text{sym}}\left(\vec{C},\vec{N}\right)=\underset{\Omega_{\text{sym}}}{\int }p\left(\vec{R}|\vec{C},\vec{N}\right)d\vec{R}.$$

and

(2.14) $$L\left(\vec{C},\vec{N}\right)=\underset{\Omega }{\int }p\left(\vec{R}|\vec{C},\vec{N}\right)d\vec{R},$$

and their ratio by $LR_{sym}(\vec{C},\vec{N})$.

To calculate these integrals, we make use of the fact that $\Omega_{sym}$ is defined by disjoint sets of inequalities ((2.3) and (2.4)), one for each triplet. (Note that a “triplet” refers equally to a triple of stimuli and the three triadic judgments among them, independent of order. For each triplet of stimuli {x,y,z}, there are three triads (x;y,z),(y;z,x), and (z;x,y), and vice-versa.) $L_{sym}(\vec{C},\vec{N})$ is therefore a product of integrals, each over a triplet of choice probabilities:

(2.15) $$LR_{\text{sym}}\left(\vec{C},\vec{N}\right)=\frac{L_{\text{sym}}\left(\vec{C},\vec{N}\right)}{L\left(\vec{C},\vec{N}\right)}=\frac{\int_{\Omega_{\text{sym}}}p\left(\vec{R}|\vec{C},\vec{N}\right)d\vec{R}}{\int_{\Omega }p\left(\vec{R}|\vec{C},\vec{N}\right)d\vec{R}}=\frac{\int_{\Omega_{\text{sym}}}p\left(\vec{C}|\vec{R},\vec{N}\right)P\left(\vec{R}\right)d\vec{R}}{\int_{\Omega }p\left(\vec{C}|\vec{R},\vec{N}\right)P\left(\vec{R}\right)d\vec{R}}\;=\frac{\prod_{T\in \text{trip}}\left(\int_{S_{\text{sym}}\left(T\right)}\prod_{i\in T}\left(R_{i}^{C_{i}}{\left(1-R_{i}\right)}^{N_{i}-C_{i}}p_{a}\left(R_{i}\right)\right)d{\vec{R}}_{T}\right)}{\prod_{k}\int_{0}^{1}\left(R_{k}^{C_{k}}{\left(1-R_{k}\right)}^{N_{k}-C_{k}}p_{a}\left(R_{k}\right)\right)dR_{k}}$$

where T ranges over the set of triplets trip,i∈T denotes the three triads in the triplet $T,{\vec{R}}_{T}$ denotes the vector of choice probabilities for these triads, and $S_{sym}(T)$ is the domain in which at least one of (2.3) and at least one of (2.4) is false for the three components of T :

(2.16) $$S_{\text{sym}}={\left[0,1\right]}^{3}\setminus \left({\left[\frac{1}{2},1\right]}^{3}\cup {\left[0,\frac{1}{2}\right]}^{3}\right)$$

Because of (2.16), each factor in the numerator of (2.15) is an integral over the cube [0,1]^3^, from which ${\left[\frac{1}{2},1\right]}^{3}$ or ${\left[0,\frac{1}{2}\right]}^{3}$ has been excluded:

(2.17) $$\underset{S_{\text{sym}}\left(T\right)}{\int }\underset{i\in T}{\prod }\left(R_{i}^{C_{i}}{\left(1-R_{i}\right)}^{N_{i}-C_{i}}p_{a}\left(R_{i}\right)\right)d{\vec{R}}_{T}=\underset{{\left[0,1\right]}^{3}}{\int }\underset{i\in T}{\prod }\left(R_{i}^{C_{i}}{\left(1-R_{i}\right)}^{N_{i}-C_{i}}p_{a}\left(R_{i}\right)\right)d{\vec{R}}_{T}\;-\underset{{\left[\frac{1}{2},1\right]}^{3}}{\int }\underset{i\in T}{\prod }\left(R_{i}^{C_{i}}{\left(1-R_{i}\right)}^{N_{i}-C_{i}}p_{a}\left(R_{i}\right)\right)d{\vec{R}}_{T}-\underset{{\left[0,\frac{1}{2}\right]}^{3}}{\int }\underset{i\in T}{\prod }\left(R_{i}^{C_{i}}{\left(1-R_{i}\right)}^{N_{i}-C_{i}}p_{a}\left(R_{i}\right)\right)d{\vec{R}}_{T},$$

With the Dirichlet prior (2.7), each factor can be written in terms of incomplete beta functions:

(2.18) $$\overset{w}{\underset{v}{\int }}R^{C}{\left(1-R\right)}^{N-C}p_{a}\left(R\right)dR=\frac{1}{B\left(a,a\right)}\overset{w}{\underset{v}{\int }}R^{C+a-1}{\left(1-R\right)}^{N-C+a-1}dR\;=\frac{B\left(w;a+C,a+N-C\right)-B\left(v;a+C,a+N-C\right)}{B\left(a,a\right)},$$

where

(2.19) $$B\left(w;a,b\right)=\overset{w}{\underset{0}{\int }}u^{a-1}{\left(1-u\right)}^{b-1}du.$$

Each factor in the denominator of (2.15) can be expressed in terms of beta functions:

(2.20) $$\overset{1}{\underset{0}{\int }}\left(R_{k}^{C_{k}}{\left(1-R_{k}\right)}^{N_{k}-C_{k}}p_{a}\left(R_{k}\right)\right)dR_{k}\;=\frac{1}{B\left(a,a\right)}\overset{1}{\underset{0}{\int }}\left(R_{k}^{C_{k}}{\left(1-R_{k}\right)}^{N_{k}-C_{k}}R_{k}^{a-1}{\left(1-R_{k}\right)}^{a-1}\right)dR_{k}\;=\frac{1}{B\left(a,a\right)}\overset{1}{\underset{0}{\int }}\left(R_{k}^{C_{k}+a-1}{\left(1-R_{k}\right)}^{N_{k}-C_{k}+a-1}\right)dR_{k}\;=\frac{B\left(a+C_{k},a+N_{k}-C_{k}\right)}{B\left(a,a\right)}.$$

Thus, the likelihood ratio in (2.15) reduces to

(2.21) $$LR_{\text{sym}}\left(\vec{C},\vec{N}\right)=\underset{T\in \text{T}}{\prod }\left(1-\underset{i\in T}{\prod }\left(1-\frac{B\left(\frac{1}{2};a+C_{i},a+N_{i}-C_{i}\right)}{B\left(a+C_{i},a+N_{i}-C_{k}\right)}\right)-\underset{i\in T}{\prod }\left(\frac{B\left(\frac{1}{2};a+C_{i},a+N_{i}-C_{i}\right)}{B\left(a+C_{i},a+N_{i}-C_{i}\right)}\right)\right).$$

For numerical reasons, many software packages (e.g., MATLAB) provide the normalized beta-function

(2.22) $$B_{\text{norm}}\left(w;a,b\right)≜\frac{B\left(w;a,b\right)}{B\left(a,b\right)}=\frac{1}{B\left(a,b\right)}\overset{w}{\underset{0}{\int }}u^{a-1}{\left(1-u\right)}^{b-1}du,$$

which recasts the result as

(2.23) $$\text{log }LR_{\text{sym}}\left(\vec{C},\vec{N}\right)\underset{T\in \text{T}}{\sum }\text{log}\left(1-\underset{i\in T}{\prod }\left(1-B_{\text{norm}}\left(\frac{1}{2};a+C_{i},a+N_{i}-C_{i}\right)\right)-\underset{i\in T}{\prod }\left(B_{\text{norm}}\left(\frac{1}{2};a+C_{i},a+N_{i}-C_{i}\right)\right)\right).$$

In sum, (2.23) tests consistency of an experimental dataset with a symmetric dis-similarity by comparing the mass of the posterior distribution of choice probabilities contained within the region $\Omega_{sym}$ consistent with the conditions (2.3) and (2.4), to the total mass. Each triplet of triadic judgments contributes an independent additive term to the log of this ratio. We therefore normalize by the number of triplets, leading to an index that quantifies the consistency of the observations with a symmetric dissimilarity:

(2.24) $$I_{\text{sym}}\left(\vec{C},\vec{N}\right)=\frac{1}{\#\left(\text{trip}\right)}\text{log }LR_{\text{sym}}\left(\vec{C},\vec{N}\right)$$

Values close to zero indicate that nearly all of the posterior distribution of choice probabilities lie within $\Omega_{\text{sym}}$; progressively more negative values indicate that the posterior shifts into its complement $\Omega \setminus \Omega_{sym}$ in which symmetry is violated.

A useful benchmark is that in the absence of any data (i.e., $\vec{C}=\vec{N}=\vec{0}$), each of the normalized beta-functions has a value of $B_{\text{norm }}\left(\frac{1}{2};a,a\right)=\frac{1}{2}$, so

(2.25) $$I_{\text{sym}}\left(\vec{0},\vec{0}\right)=\frac{1}{\#\left(\text{trip}\right)}\text{log}\underset{T\in \text{ trip }}{\prod }\left(1-{\left(1-\frac{1}{2}\right)}^{3}-{\left(\frac{1}{2}\right)}^{3}\right)=\text{log}\frac{3}{4}\approx -0.2877,$$

independent of the Dirichlet parameter a. Thus, values of $I_{sym}(\vec{C},\vec{N})$ greater than $log\frac{3}{4}$ are more consistent with symmetry than an index derived from a large number of choice probabilities drawn randomly from the prior. Also note that deviations from this *a priori* value can only be driven by triplets in which there are observations for at least two of the triads. This follows from the fact that $B_{\text{norm }}\left(\frac{1}{2};a,a\right)=\frac{1}{2}$, so that if only one triad k∈T has a nonzero number of observations,

(2.26) $$1-\underset{i\in T}{\prod }\left(1-B_{\text{norm}}\left(\frac{1}{2};a+C_{i},a+N_{i}-C_{i}\right)\right)-\underset{i\in T}{\prod }\left(B_{\text{norm}}\left(\frac{1}{2};a+C_{i},a+N_{i}-C_{i}\right)\right)\;=1-\frac{1}{2}•\frac{1}{2}•\left(1-B_{\text{norm}}\left(\frac{1}{2};a+C_{k},a+N_{k}-C_{k}\right)\right)-\frac{1}{2}•\frac{1}{2}•B_{\text{norm}}\left(\frac{1}{2};a+C_{k},a+N_{k}-C_{k}\right)\;=\frac{3}{4}.$$

This makes intuitive sense: we can only make inferences about the structure of the dis-similarity judgments if there is experimental data about more than one triad within a triplet. With data about only one triad, knowing the sign of the comparison is useless since this sign is arbitrarily determined by how the triad is labeled, i.e., (r;x,y) vs. (r;y,x).

Finally, we note that this analysis focuses on a condition that is necessary for symmetry, but is not sufficient. The chain of inequalities of eq. (2.1) the simplest of a series of necessary conditions: more generally, for any n-cycle $\left(a_{1},a_{2},\ldots ,a_{n}\right)$, there is a set of inequalities

(2.27) $$\begin{array}{c} D\left(a_{1},a_{2}\right)<D\left(a_{1},a_{n}\right)\\ D\left(a_{n},a_{1}\right)<D\left(a_{n},a_{n-1}\right)\\ \vdots \\ D\left(a_{2},a_{3}\right)<D\left(a_{2},a_{1}\right) \end{array}\}$$

for which at least one must be false. Otherwise (generalizing (2.2)), there would be a contradiction:

(2.28) $$D(a_{1},a_{2})<D(a_{1},a_{n})=D(a_{n},a_{1})<D(a_{n},a_{n-1})=D(a_{n-1},a_{n})<\cdots =D(a_{2},a_{3})<D(a_{2},a_{1}),$$

Hence, at least one of

(2.29) $$\begin{array}{c} R\left(a_{1};a_{2},a_{n}\right)>1/2\\ R\left(a_{n};a_{1},a_{n-1}\right)>1/2\\ \vdots \\ R\left(a_{2};a_{3},a_{1}\right)>1/2 \end{array}\}$$

must be false for a symmetric dis-similarity. It is possible to construct scenarios in which this criterion is violated, but the triplet criteria (2.27) hold.

These conditions can be analyzed in a manner analogous to the triplet conditions above, but we do not pursue this analysis here: these more elaborate conditions exclude progressively smaller portions of the choice probability space, as they rely on a conjunction of progressively more inequalities. These additional conditions are also not independent of each other, since (for n≥4), the triads in (2.29) that correspond to different n-cycles may be partially overlapping with each other. Such overlaps prevent the factorization that facilitated exact evaluation of the likelihood ratio integrals.

### Assessing ultrametric structure

The motivation for the next index begins with the observation that consistency with a symmetric dis-similarity guarantees consistency with a metric-space structure (Appendix 1). Since symmetric dis-similarities guarantee consistency with a metric-space structure, it is therefore natural to ask whether the dis-similarities have further properties consistent with specific kinds of metric spaces.

Ultrametric spaces (Semmes, 2007) are one important such kind, as they abstract the notion of a hierarchical organization. Points in an ultrametric space correspond to the terminal nodes of a tree, and the distance between two points corresponds to the height of their first common ancestor. Formally, a distance d is said to satisfy the ultrametric inequality if, for any three points x,y, and z,

(3.1) d(x,y)≤max(d(x,z),d(y,z)),

a condition that implies the triangle inequality (Appendix 1). Essentially, (3.1) states that any triangle is isosceles, with the two equal sides no shorter than the third.

#### Necessary and sufficient conditions

To determine the extent to which a set of responses is consistent with the ultrametric inequality, we first restate (3.1) in terms of dis-similarities and distances:

(3.2) D(x,y)≤max(D(x,z),D(y,z)).

Since (Appendix 1, eq. (6.6)) dis-similarities can always be transformed into distances via a monotonic transformation, consistency of the dis-similarity structure with (3.2) is equivalent to consistency with distances that satisfy the ultrametric property (3.1).

We now recast (3.2) in terms of choice probabilities. Since the conditions need to apply to the three points x,y, and z taken in any order, (3.2) means that among D(x,y),D(y,z) and D(z,x), two must be equal and no less than the third. Writing $R_{1}≜R(x;y,z),R_{2}≜R(y;z,x)R_{3}≜R(z;x,y)$, this means that at least one of the following must hold:

(3.3) $$D\left(x,y\right)\leq D\left(z,x\right)=D\left(y,z\right)\Leftrightarrow R_{1}\geq \frac{1}{2},R_{2}\leq \frac{1}{2},R_{3}=\frac{1}{2}\;D\left(y,z\right)\leq D\left(x,y\right)=D\left(z,x\right)\Leftrightarrow R_{1}=\frac{1}{2},R_{2}\geq \frac{1}{2},R_{3}\leq \frac{1}{2}\;D\left(z,x\right)\leq D\left(y,z\right)=D\left(x,y\right)\Leftrightarrow R_{1}\leq \frac{1}{2},R_{2}=\frac{1}{2},R_{3}\geq \frac{1}{2}.$$

We denote the region of $\left(R_{1},R_{2},R_{3}\right)$-space defined by (3.3) as $S_{umi}$. Just as we had measured consistency with symmetry by determining the fraction of the posterior distribution of choice probabilities that lies in $S_{sym}$, here we seek to measure consistency with the ultrametric condition by determining to what extent the posterior distribution of choice probabilities lies in $S_{umi}$.

#### Likelihood ratio calculation and an index

A direct application of the machinery developed in the previous section runs into an immediate difficulty: the conditions (3.3) are only satisfied on a set of measure zero, when at least one of the $R_{i}$ are exactly equal to $\frac{1}{2}$. So a Bayesian analysis that begins with a Dirichlet prior will always lead to a likelihood ratio of zero, independent of the data: because the Dirichlet prior is continuous, any posterior derived from via Bayes rule will never have a discrete mass at $\frac{1}{2}$, as would be required to satisfy (3.3).

It is nevertheless possible to capture the spirit of ultrametric behavior in a rigorous way, and at the same time, address a way in which the Dirichlet prior may be unrealistic. To do this, we recognize that for some triads, it may be appropriate to model the underlying choice probability as exactly $\frac{1}{2}$, corresponding to stimuli for which there is no basis for comparison: is a toothbrush or a mountain more similar to an orange? But we don’t know, *a priori*, how many of the triads have this property. To take this into account, we generalize the prior for each choice probability to be a sum of two components: one component is the Dirichlet prior used above (2.7), normalized to 1-h; the second component is a point mass at $\frac{1}{2}$, normalized to h :

(3.4) $$p_{a,h}\left(R\right)≜\left(1-h\right)p_{a}\left(R\right)+h\delta \left(R-\frac{1}{2}\right)=\frac{1-h}{B\left(a,a\right)}R^{a-1}{\left(1-R\right)}^{a-1}+h\delta \left(R-\frac{1}{2}\right).$$

With this prior, we can then determine the likelihood ratio as a function of h. For small values of h, the likelihood ratio will be proportional to h, since the mass in the prior at $\frac{1}{2}$ is proportional to h. The proportionality as h→0 thus serves as an index of consistency with the ultrametric property. An alternative approach (not taken here) is that if the experimental dataset suggest that a prior $p_{a,h}(R)$ with h>0 is a substantially better fit to the distribution of choice probabilities than $p_{a,0}(R)$, this prior can be used directly to calculate a likelihood ratio, and the best-fitting value of h then provides an additional descriptor of the dataset.

To implement this strategy, we write the likelihood ratio as

(3.5) $$LR_{\text{umi}}\left(\vec{C},\vec{N};h\right)=\frac{U_{\text{umi}}\left(\vec{C},\vec{N};h\right)}{U_{\text{sym}}\left(\vec{C},\vec{N};h\right)}.$$

where the numerator is a likelihood equal to the integral over choice probabilities consistent with the ultrametric inequality (and a symmetric dis-similarity), and the denominator is a likelihood equal to the integral over choice probabilities consistent with a symmetric dis-similarity but without the ultrametric constraint. As above, because the triads form independent triplets, both numerator and denominator can be factored into a product of terms $U_{umi}\left({\vec{C}}_{T},{\vec{N}}_{T};h\right)$ or $U_{sym}\left({\vec{C}}_{T},{\vec{N}}_{T};h\right)$, one for each triplet T∈trip. These terms are of the form

(3.6) $$U\left({\vec{C}}_{T},{\vec{N}}_{T};h\right)=\underset{{\left[0,1\right]}^{k}}{\int }V\left(\text{sgn}\left(R_{1}-\frac{1}{2}\right),\cdots ,\text{sgn}\left(R_{k}-\frac{1}{2}\right)\right)R_{1}^{C_{1}}{\left(1-R_{1}\right)}^{N_{1}-C_{1}}•\cdots •R_{k}^{C_{k}}{\left(1-R_{k}\right)}^{N_{k}-C_{k}}•p_{a,h}\left(R_{1}\right)•\cdots •p_{a,h}\left(R_{k}\right)dR_{1}\cdots dR_{k},$$

where $V\left(\sigma_{1},\cdots ,\sigma_{k}\right)$, which is 0 or 1, defines the space of choice probabilities that contributes to the integral for the triads in T. Since consistency of choice probabilities with the ultrametric condition or symmetry depends only on their rank order, V only depends on whether the choice probabilities are less than, equal to, or greater than $\frac{1}{2}$. In (3.6), k=3 (for the three triads within a triplet); the analysis of additive trees will require need k=6.

For the numerator of (3.5), we incorporate the ultrametric conditions (3.3) into $V=V_{\text{umi}}$ :

(3.7) $$V_{\text{umi}}\left(+1,-1,0\right)=1,\text{ corresponding to }R_{1}>\frac{1}{2},R_{2}<\frac{1}{2},R_{3}=\frac{1}{2}\;V_{\text{umi}}\left(0,+1,-1\right)=1,\text{ corresponding to }R_{1}=\frac{1}{2},R_{2}\geq \frac{1}{2},R_{3}\leq \frac{1}{2}\;V_{\text{umi}}\left(-1,0,+1\right)=1,\text{ corresponding to }R_{1}\leq \frac{1}{2},R_{2}=\frac{1}{2},R_{3}\geq \frac{1}{2}\;V_{\text{umi}}\left(0,0,0\right)=1,\text{ corresponding to }R_{1}=\frac{1}{2},R_{2}=\frac{1}{2},R_{3}=\frac{1}{2}.$$

All other values of $V_{umi}(\vec{\sigma })$ are zero, since either they don’t correspond to any of the conditions (3.3), or to exactly two of those conditions. This latter is impossible, as it would require two equalities and one strict inequality among the ‘s.

For the denominator of (3.5), we incorporate the symmetry conditions of the previous section into $V=V_{\text{sym}}$, adding an explicit consideration of the borderline cases. Thus, $V_{\text{sym}}=1$ for the following arguments: (i) When no arguments are zero, both signs must be represented among the arguments. This corresponds to the requirement that at least one of (2.3) and at least one of (2.4) are false. (ii) When one argument is zero, for all arguments at which $V_{umi}(\vec{\sigma })=1$ in (3.7) (corresponding to an isosceles triangle, with the equal sides larger than the third), and also all of the values for which $V_{umi}(-\vec{\sigma })=1$ in (3.7) (corresponding to an isosceles triangle, with the equal sides smaller than the third). (iii) $V_{sym}(0,0,0)=1$ (corresponding to an equilateral triangle):

(3.8) $$\left(i\right)V_{\text{sym}}\left(\pm 1,\pm 1,\mp 1\right)=V_{\text{sym}}\left(\pm 1,\mp 1,\pm 1\right)=V_{\text{sym}}\left(\mp 1,\pm 1,\pm 1\right)=1\;\left(ii\right)V_{\text{sym}}\left(\pm 1,\mp 1,0\right)=V_{\text{sym}}\left(0,\pm 1,\mp 1\right)=V_{\text{sym}}\left(\mp 1,0,\pm 1\right)=1\;\left(iii\right)V_{\text{sym}}\left(0,0,0\right)=1.$$

Equivalently, $V_{sym}$ is nonzero when, and only when, either (a) arguments include both positive and negative signs, or (b) all arguments are zero.

To establish the behavior of the likelihood ratio (3.5) as h→0, we use (3.4) to isolate the dependence of integrals (3.6) on h. This is a polynomial:

(3.9) $$U\left({\vec{C}}_{T},{\vec{N}}_{T};h\right)=\underset{\vec{\sigma }}{\sum }h^{Z\left(\vec{\sigma }\right)}{\left(1-h\right)}^{k-Z\left(\vec{\sigma }\right)}V\left(\sigma_{1},\cdots ,\sigma_{k}\right)W\left(\sigma_{1};C_{1},N_{1}\right)•\cdots •W\left(\sigma_{k};C_{k},N_{k}\right),$$

where the sum is over all $3^{k}$ assignments of the elements of $\vec{\sigma }=\left(\sigma_{1},\cdots ,\sigma_{k}\right)$ to $\left\{-1,0,+1\},Z(\vec{\sigma }\right)$ is the number of entries in $\vec{\sigma }$ that are equal to zero (each such entry incurring a factor of h), and W(σ;C,N) is the integral of the prior (3.4), weighted by the experimental data, over one segment of the domain:

(3.10) $$W\left(\sigma ;C,N\right)=\{\begin{array}{l} \overset{\frac{1}{2}}{\underset{0}{\int }}R^{C}{\left(1-R\right)}^{N-C}p_{a}\left(R\right)dR,\sigma =-1\\ \overset{\frac{1}{2}+\varepsilon }{\underset{\frac{1}{2}-\varepsilon }{\int }}R^{C}{\left(1-R\right)}^{N-C}\delta \left(R-\frac{1}{2}\right)dR,\sigma =0,\\ \overset{1}{\underset{\frac{1}{2}}{\int }}R^{C}{\left(1-R\right)}^{N-C}p_{a}\left(R\right)dR,\sigma =+1 \end{array}$$

These evaluate to

(3.11) $$W\left(\sigma ;C,N\right)=\{\begin{array}{c} \frac{1}{B\left(a,a\right)}B\left(\frac{1}{2};a+C,a+N-C\right),\sigma =-1\\ \frac{1}{2^{N}},\sigma =0\\ \frac{1}{B\left(a,a\right)}\left(1-B\left(\frac{1}{2};a+C,a+N-C\right)\right),\sigma =+1 \end{array},$$

Consequently,

(3.12) $$U\left({\vec{C}}_{T},{\vec{N}}_{T};0\right)=\underset{Z\left(\vec{\sigma }\right)=0}{\sum }V\left(\sigma_{1},\cdots ,\sigma_{k}\right)W\left(\sigma_{1};C_{1},N_{1}\right)•\cdots •W\left(\sigma_{k};C_{k},N_{k}\right),$$

(3.13) $$\frac{d}{dh}U\left({\vec{C}}_{T},{\vec{N}}_{T};h\right)=\underset{\vec{\sigma }}{\sum }\left(Z\left(\vec{\sigma }\right)h^{Z\left(\vec{\sigma }\right)-1}{\left(1-h\right)}^{k-Z\left(\vec{\sigma }\right)}-\left(k-Z\left(\vec{\sigma }\right)\right)h^{Z\left(\vec{\sigma }\right)}{\left(1-h\right)}^{k-Z\left(\vec{\sigma }\right)-1}\right)•\;V\left(\sigma_{1},\cdots ,\sigma_{k}\right)W\left(\sigma_{1};C_{1},N_{1}\right)•\cdots •W\left(\sigma_{k};C_{k},N_{k}\right),$$

and

(3.14) $$\frac{d}{dh}U\left({\vec{C}}_{T},{\vec{N}}_{T};0\right)=\underset{Z\left(\vec{\sigma }\right)=1}{\sum }V\left(\sigma_{1},\cdots ,\sigma_{k}\right)•W\left(\sigma_{1};C_{1},N_{1}\right)•\cdots •W\left(\sigma_{k};C_{k},N_{k}\right)+-k\underset{Z\left(\vec{\sigma }\right)=0}{\sum }V\left(\sigma_{1},\cdots ,\sigma_{k}\right)•W\left(\sigma_{1};C_{1},N_{1}\right)•\cdots •W\left(\sigma_{k};C_{k},N_{k}\right).$$

For the numerator of (3.5), $U_{umi}\left({\vec{C}}_{T},{\vec{N}}_{T};0\right)=0$, since the nonzero values of $V_{umi}(\vec{\sigma })$ all have $Z(\vec{\sigma })\geq 1$; thus, the small- h behavior is proportional to (3.14). For the denominator of (3.5), $U_{\text{sym }}\left({\vec{C}}_{T},{\vec{N}}_{T};0\right)$ is nonzero since $V_{\text{sym }}(\vec{\sigma })=1$ for several triplets of nonzero arguments (those that include positive and negative signs). Thus, for small h, the likelihood ratio (3.5) is proportional to h-- and this proportionality tells us to what extent adding a small amount of mass to the prior at $R=\frac{1}{2}$ captures triplets of choice probabilities that are consistent with the ultrametric property.

This analysis motivates an index of the extent to which a set of observations supports a model in which dis-similarities are consistent with an ultrametric model:

(3.15) $$I_{\text{umi}}\left(\vec{C},\vec{N}\right)=\frac{1}{\#\left(\text{trip}\right)}{\text{lim}}_{h\to 0}\left(\text{log }LR_{\text{umi}}\left(\vec{C},\vec{N};h\right)\right)-\text{log }h,$$

As is the case with the index of symmetry $I_{sym}$ (eq. (2.24)), a useful benchmark is the a priori value, $I_{umi}(0,0)$. From (3.11),

(3.16) $$W\left(\sigma ;0,0\right)=\{\begin{array}{cc} \frac{1}{2}, & \sigma =\pm 1\\ 1, & \sigma =0 \end{array}.$$

There are three nonzero contributors to $V_{umi}$ with $Z(\vec{\sigma })=1$ (see (3.7)), so this yields

(3.17) $$U_{\text{umi}}\left(0,0;h\right)=\frac{3}{2^{2}}h+O\left(h^{2}\right)$$

from (3.14). There are six nonzero contributors to $V_{\text{sym}}$ with $Z(\vec{\sigma })=0$ (any set of arguments that includes both signs), so (3.12) and (3.16) yield

(3.18) $$U_{\text{sym}}\left(0,0;0\right)=\frac{6}{2^{3}}\text{.}$$

Since each of the triplets contributes an equal factor to the likelihood ratio,

(3.19) $$I_{\text{umi}}\left(\vec{C},\vec{N}\right)={\text{lim}}_{h\to 0}\left(\text{log}\left(\frac{\frac{3}{4}h+O\left(h^{2}\right)}{\frac{3}{4}}\right)-\text{log }h\right)=0.$$

In sum, the index $I_{umi}(\vec{C},\vec{N})$ (eq. (3.15)) evaluates whether an experimental set of dis-similarity responses is consistent with an ultrametric model, and does so in a way that recognizes the intrinsic limitation that experimental data can never show that a choice probability is exactly $\frac{1}{2}$. If the index is greater than 0, the observed data are more consistent with an ultrametric model than a set of unstructured choice probabilities; values less than 0 indicate progressively greater deviations from an ultrametric model.

### Assessing additive tree structure

Like the ultrametric model, the additive similarity tree model (Sattath & Tversky, 1977) is metric space model that places constraints on the properties of the distance, but these constraints are less-restrictive than the constraints of the ultrametric model (Appendix 2). In this model, here referred to as “addtree,” the distance between two points is determined by a graph that has a tree structure, in which each link has a specified nonzero weight. The distance between two points is given by the total weight of the path that connects the points. Because of the requirement that the graph is a tree structure, there are no loops – and this places constraints on the inter-relationships of the distances.

Here, we determine the extent to which the dis-similarities implied by a set of triadic judgments can be monotonically transformed into the distances in an addtree model.

The starting point is a necessary and sufficient condition for distances in a metric space to be consistent with an addtree structure (Buneman, 1974; Dobson, 1974; Sattath & Tversky, 1977). This condition, known as the “four-point condition,” is that given any four points u,v,w, and x,

(4.1) $$\text{None of the three quantities}\left\{\begin{array}{c} d\left(u,v\right)+d\left(w,x\right)\\ d\left(u,w\right)+d\left(v,x\right)\\ d\left(u,x\right)+d\left(v,w\right) \end{array}\right\}\text{is strictly greater than the other two}.$$

Put another way, of the three pairwise sums in eq. (4.1), two must be equal, and larger than the third. Appendix 2 shows that this condition is weaker than the ultrametric inequality and stronger than the triangle inequality, and that a one-dimensional arrangement of points is always consistent with an addtree model.

Since the four-point condition is based on adding distances, we cannot apply it directly to dis-similarities – as distances are linked to dis-similarity via an unknown monotonic function. Instead, we frame a condition on the dis-similarities that is necessary for the four-point inequality to hold.

#### Necessary conditions

Since the four-point condition asserts that, among the three ways of pairing four points, none can result in a total distance that is strictly greater than the other two, it is forced to fail if the three pairwise-summed distances are distinct. If this is the case, we can relabel the points so that

(4.2) d(u,v)+d(w,x)>d(u,w)+d(v,x)>d(u,x)+d(v,w).

If (4.2) holds, (4.1) cannot. So failures of at least one of the inequalities in (4.2) is necessary for the addtree model, and consequently, inequalities among dis-similarities that guarantee the inequalities among the distances in (4.2) rule out the addtree model.

For example, the inequalities in (4.2) are forced to hold if the three first terms and the three second terms in each of the its sums are in descending order:

(4.3) d(u,v)>d(u,w)>d(u,x) and d(w,x)>d(v,x)>d(v,w).

Since the distances are monotonically related to the dis-similarities, (4.3) is equivalent to

(4.4) D(u,v)>D(u,w)>D(u,x) and D(w,x)>D(v,x)>D(v,w).

Thus, (4.4), which does not rely on adding dis-similarities or distances, suffices to rule out the addtree model.

**Figure 1.**
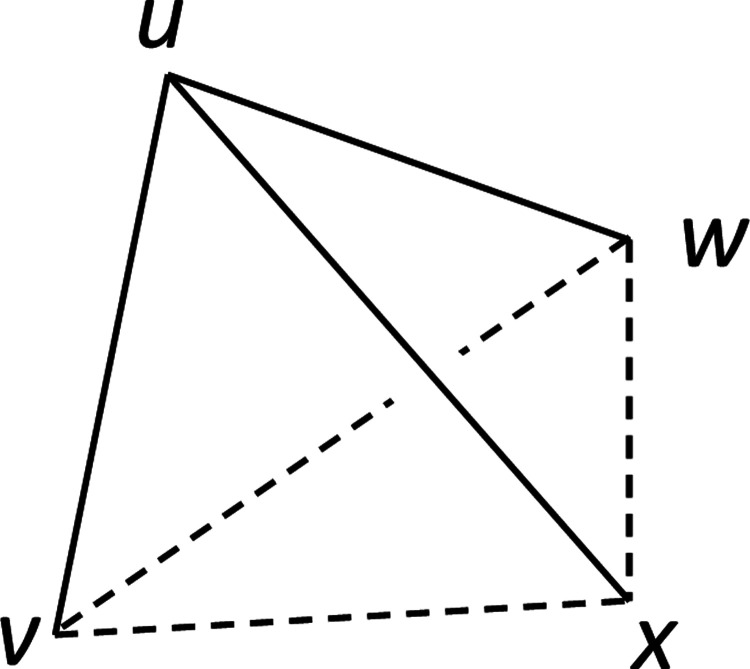
A tent of dis-similarities, consisting of a tripod, the three dis-similarities involving the vertex u (solid lines), and a base, the three opposite dis-similarities spanning the vertices v,w, and x (dashed lines).

It is useful to think of the two parts of (4.4) geometrically, with the four points $\left\{u,v,w,x\right\}$ arranged in a tetrahedron (Figure 1). The first part of (4.4) compares dis-similarities that pair one vertex u with the other three vertices; we call this set of dis-similarities a “tripod”. The second part of (4.4) compares the dis-similarities of the edges of the triangle on the face opposite to u; we call these dis-similarities the “base”. The combination of a tripod and a base is a “tent” -a quadruple of points in which one of them is distinguished.

Assuming all dis-similarities are distinct, we can relabel the points v,w, and x so that the dis-similarities involving u are in descending order. Thus, (4.4) can be restated simply: the rank order of dis-similarities of the tripod, and the rank order of dis-similarities of the opposite edges in the base, cannot be identical.

This viewpoint reveals other ways that rank-orders of dis-similarities can force (4.2): whenever the longest side of a tripod and the longest side of a base are opposite each other. When this happens, the leftmost pair of (4.2) is necessarily larger than each of the other two pairs, and the four-point condition (4.1) cannot hold. That is, for a tent with vertex z and base {a,b,c}, the following conjunction rules out the addtree model:

(4.5) D(z,c)>D(z,b) and D(z,c)>D(z,a) and D(a,b)>D(c,a) and D(a,b)>D(b,c).

The addtree model is also ruled out if some of the inequalities in (4.5) are non-strict: D(z,c)>D(z,a) can be replaced by D(z,c)≥D(z,a) provided that D(a,b)>D(b,c) remains strict (and vice-versa), and similarly for D(z,c)>D(z,b) and D(a,b)>D(c,a). Note that this conjunction only involves choice probabilities within the tripod, or within the base.

We show in Appendix 3 that if the conjunction (4.5) is false for the dis-similarities among four points and all of their relabelings, then there is a monotonic transformation d=F(D) of the dis-similarities into distances, for which the distances satisfy the four-point condition. That is, falsity of the conjunction (4.5) is necessary for an addtree model and sufficient for an addtree model among the four points. This stops short of showing that falsification of the conjunction (4.5) for all quadruplets is sufficient for a global addtree model, as the argument in Appendix 3 only identifies a monotonic transformation of the dis-similarities among individual four-point subsets. Appendix 3 does not show that the monotonic transformations needed for each quadruple of points can be made in a globallyconsistent way, though we do not have examples to the contrary.

#### Likelihood ratio calculation and an index

We now formulate an index that measures the extent to which the choice probabilities underlying a set of observations corresponds to dis-similarities that falsify the conjunction (4.5). This index therefore reflects the likelihood that the choice probabilities fulfill a condition that is necessary for an addtree model.

At first, we consider an index closely analogous to (2.24): a log-likelihood ratio, averaged over tents, comparing the mass of choice probabilities that is within the region in which the conjunction (4.5) is falsified, to the mass that is merely consistent with a symmetric dis-similarity of the choice probabilities that make up the tent:

(5.1) $$I_{\text{addtree}}\left(\vec{C},\vec{N};h\right)=\frac{1}{\#\left(\text{tent}\right)}\text{log}\left(\frac{U_{\text{addtree}}\left(\vec{C},\vec{N};h\right)}{U_{\text{symtent}}\left(\vec{C},\vec{N};h\right)}\right),$$

where tent is the set of tents.

This formulation, however, is problematic: in contrast to the indices for symmetry and ultrametric structure, which were averages over triplets, this is an average over tents. Distinct triplets have non-overlapping triads, but for distinct tents, triads may overlap. For example, a tent with vertex z and base {a,b,c} has a triad (z;a,b), and this triad is shared with the tent with vertex z and base {a,b,q}, and with the tent with vertex y and base {z,a,b}. Thus, the previous factorization of the likelihood integrals into low-dimensional pieces does not apply to the numerator of (5.1).

We therefore replace (5.1) by a heuristic approximation, in which we ignore this overlap:

(5.2) $$I_{\text{addtree}}^{\text{'}}\left(\vec{C},\vec{N};h\right)=\frac{1}{\#\left(\text{tent}\right)}\underset{T\in \text{tent}}{\sum }\text{log}\left(\frac{U_{\text{addtree}}\left({\vec{C}}_{T},{\vec{N}}_{T};h\right)}{U_{\text{syment}}\left({\vec{C}}_{T},{\vec{N}}_{T};h\right)}\right).$$

$I_{\text{addtree}}^{'}$ is no longer a log likelihood ratio, but will still express the extent to which the data are consistent with a necessary condition for the addtree model. The approximation amounts to considering each tent as providing independent evidence concerning this consistency.

The numerator and denominator of (5.2) have the same form as (3.6), so it suffices to specify the values of $V\left(sgn\left(R_{1}-\frac{1}{2}\right),\cdots ,sgn\left(R_{6}-\frac{1}{2}\right)\right)$ for the numerator $\left(V_{\text{addtree}}\right)$ and denominator $V_{\text{symtent}}$. For definiteness, given a tent with z at the vertex and {a,b,c} at the base, we specify the six choice probabilities needed to compute V as follows: for the tripod component, $R_{1}≜R(z;b,c)$, $R_{2}≜R(z;c,a),R_{3}≜R(z;a,b);$ for the base, $R_{4}≜R(a;b,c),R_{5}≜R(b;c,a),R_{6}≜R(c;a,b)$. All of these (and no others) enter into the condition that (4.5) is falsified for this tent: the choice probabilities $R_{1},R_{2},R_{4}$, and $R_{5}$ are explicit in (4.5), all of the $R_{i}$ are used equally as the base elements {a,b,c} are permuted. Since V has six arguments, each of which can take on any of three values {-1,0,1}, there are $3^{6}=729$ values to specify.

For $V_{\text{addree}}$, it is simplest to specify these values algorithmically. For a set of choice probabilities to be consistent with the addtree model (i.e., for $V_{\text{addree}}=1$), the conjunction (4.5) cannot hold for any of the permutations of {a,b,c}. Since (4.5) is symmetric under interchange of a and b, it suffices to consider the cyclic permutations. So the region of $[0,1{]}^{6}$ in which $V_{\text{addree}}=1$ is the intersection of the region that falsifies the conjunction (4.5), which we denote $V_{\text{addree}}^{\left[c\right]}$, and the regions that falsify it after cyclic permutation of (a,b,c), which we denote $V_{\text{addrree}}^{\left[a\right]}$ and $V_{\text{addrree}}^{\left[b\right]}$. Additionally, $V_{\text{addtree}}=0$ for sets of choice probabilities that are inconsistent with a symmetric dis-similarity. Thus,

(5.3) $$V_{\text{addtree}}=V_{\text{addtree}}^{\left[a\right]}V_{\text{addtree}}^{\left[b\right]}V_{\text{addtre }}^{\left[c\right]}V_{\text{symten }}.$$

$V_{\text{addrree}}^{\left[c\right]}=1$ except when all of the inequalities of (4.5) hold, or, as noted following that equation, when D(z,c)>D(z,a) or D(a,b)>D(b,c) (but not both) is replaced by equality, and D(z,c)>D(z,b) or D(a,b)>D(c,a) (but not both) is replaced by equality:

(5.4) $$V_{\text{addtree}}^{\left[c\right]}\left(+\tau_{1},-\tau_{2},\sigma_{3},-\tau_{4},+\tau_{5},\sigma_{6}\right)=0\;\text{for }\left(\tau_{1},\tau_{4}\right)\text{ and }\left(\tau_{2},\tau_{5}\right)\in \left\{\left(1,1\right),\left(1,0\right),\left(0,1\right)\right\};\sigma_{3}\text{ and }\sigma_{6}\in \left\{-1,0,+1\right\}’$$

Here, the paired $\tau_{i}$ ‘s -- not both of which can be zero -- handle the allowed equalities, and the σ ‘s handle the lack of a dependence on the third and sixth arguments. $V_{\text{addrree}}^{\left[a\right]}$ and $V_{\text{addtree}}^{\left[b\right]}$ are then determined by cyclic permutation:

(5.5) $$V_{\text{addtree}}^{\left[a\right]}\left(\sigma_{1},\sigma_{2},\sigma_{3},\sigma_{4},\sigma_{5},\sigma_{6}\right)=V_{\text{addtree}}^{\left[c\right]}\left(\sigma_{2},\sigma_{3},\sigma_{1},\sigma_{5},\sigma_{6},\sigma_{4}\right)\;V_{\text{addtree}}^{\left[b\right]}\left(\sigma_{1},\sigma_{2},\sigma_{3},\sigma_{4},\sigma_{5},\sigma_{6}\right)=V_{\text{addtree}}^{\left[c\right]}\left(\sigma_{3},\sigma_{1},\sigma_{2},\sigma_{6},\sigma_{4},\sigma_{5}\right).$$

$V_{\text{symtent}}$ occurs both as a factor in the numerator (5.3) and alone in the denominator. The three triads in the base, which only depend on dis-similarities between the elements of the triplet {a,b,c}, so the choice probabilities consistent with symmetry are determined by $V_{\text{sym}}\left(\sigma_{4},\sigma_{5},\sigma_{6}\right)$ (eq. (3.8)). The three triads in the tripod are comparisons between D(z,a),D(z,b), and D(z,c). While these are unconstrained by symmetry, they must be self-consistent. That is, all of the inequalities:

(5.6) $$\begin{array}{r} D\left(z,a\right)<D\left(z,b\right)\\ D\left(z,b\right)<D\left(z,c\right)\\ D\left(z,c\right)<D\left(z,a\right) \end{array}\}$$

cannot hold, nor can they all hold if all signs of comparison are inverted. This is precisely the constraints of (2.3) and (2.4), so it is captured by $V_{\text{sym}}\left(\sigma_{1},\sigma_{2},\sigma_{3}\right)$ (eq. (3.8)). Thus,

(5.7) $$V_{\text{symtent}}\left(\sigma_{1},\sigma_{2},\sigma_{3},\sigma_{4},\sigma_{5},\sigma_{6}\right)=V_{\text{sym}}\left(\sigma_{1},\sigma_{2},\sigma_{3}\right)V_{\text{sym}}\left(\sigma_{4},\sigma_{5},\sigma_{6}\right).$$

In sum, the index (5.2) is specified by

(5.8) $$U_{\text{addtree}}\left({\vec{C}}_{T},{\vec{N}}_{T};h\right)=\underset{{\left[0,1\right]}^{6}}{\int }V_{\text{addtree}}\left(\text{sgn}\left(R_{1}-\frac{1}{2}\right),\cdots ,\text{sgn}\left(R_{6}-\frac{1}{2}\right)\right)R_{1}^{C_{1}}{\left(1-R_{1}\right)}^{N_{1}-C_{1}}•\cdots •R_{6}^{C_{6}}{\left(1-R_{6}\right)}^{N_{6}-C_{6}}•p_{a,h}\left(R_{1}\right)•\cdots •p_{a,h}\left(R_{6}\right)dR_{1}\cdots dR_{k}$$

and

(5.9) $$U_{\text{symtent}}\left({\vec{C}}_{T},{\vec{N}}_{T};h\right)=\underset{{\left[0,1\right]}^{6}}{\int }V_{\text{symtent}}\left(\text{sgn}\left(R_{1}-\frac{1}{2}\right),\cdots ,\text{sgn}\left(R_{6}-\frac{1}{2}\right)\right)R_{1}^{C_{1}}{\left(1-R_{1}\right)}^{N_{1}-C_{1}}•\cdots •R_{6}^{C_{6}}{\left(1-R_{6}\right)}^{N_{6}-C_{6}}•p_{a,h}\left(R_{1}\right)•\cdots •p_{a,h}\left(R_{6}\right)dR_{1}\cdots dR_{k},$$

where $V_{\text{addree}}$ and $V_{\text{symtent}}$ are given by eqs. (5.3) and (5.7).

As a benchmark, we calculate the value of $I_{\text{addree}}^{'}$ based on the prior alone, for h=0. The two instances of $V_{sym}$ in its denominator (eq. (5.7)) each contribute a factor of $\frac{3}{4}$ (for each, six of 2^3^ sign combinations are included, as in the calculation of (2.25)). In the numerator, by direct enumeration, 24 of $2^{6}$ sign combinations are nonzero. So

(5.10) $$I_{\text{addrree}}^{\prime }\left(0,0;0\right)=\text{log}\left(\frac{\frac{24}{64}}{\frac{3}{4}•\frac{3}{4}}\right)=\text{log}\frac{2}{3}\sim -0.4055.$$

## Example Applications

Here we demonstrate the present approach via application to sample datasets from three psychophysical experiments, encompassing two methods for acquiring similarity judgments and spanning low- and high-level visual domains.

### Methods

The first two experiments (“textures” and “faces”) make use of the method of Waraich et al. (Waraich & Victor, 2022): on each trial, participants rank the eight comparison stimuli $c_{1},\ldots ,c_{8}$, in order of similarity to a central reference r. These rank-orderings are then interpreted as a set of similarity judgments: ranking $c_{i}$ as more similar than $c_{j}$ to the reference r is interpreted as a triadic judgment that $D\left(c_{j},r\right)>D\left(c_{i},r\right)$. Data are accumulated across all trials in which $c_{i}$ and $c_{j}$ are presented along with the reference r, leading to an estimate of $R_{obs}\left(r;c_{i},c_{j}\right)$. Stimulus sets consisted of 24 or 25 items (described in detail with Results below), and 10 sessions of 100 trials each are presented. On each trial, stimuli are randomly chosen to be the reference or the comparison stimuli. As there were 10 sessions of 100 self-paced trials each and each trial yielded $\left(\begin{array}{l} 8\\ 2 \end{array}\right)=28$ triadic judgments, each participant’s dataset contained 28,000 triadic judgments.

For the “textures” and “faces” datasets (described in detail below), stimuli were generated in MATLAB, and were displayed and sequenced using open-source PsychoPy software on a 22-inch LCD screen (Dell P2210, resolution 1680×1050, color profile D65, mean luminance 52 cd/m^2^). The display was viewed binocularly from a distance of 57 cm. The visual angle of the stimulus array was 24 degrees; each stimulus (a texture patch or a face) subtended 4 degrees. Tallying of responses and multidimensional scaling as described in (Waraich & Victor, 2022) was carried out via Python scripts. Computation of the indices and visualization was carried out in MATLAB using code that is publicly available at https://github.com/jvlab/simrank.

The third experiment (“brightness”) uses an odd-one-out paradigm. On each trial, three stimuli are presented, each consisting of a central disk, drawn from one of eight luminances, and an annular surround. The surround was either of minimal or maximal luminance, and was perceived as black or white, respectively. The participant is asked to judge the brightness of the central disk, and to choose which of the three is the outlier. We interpret selection of a stimulus $x_{j}$ out of a triplet $\left\{x_{j},x_{k},x_{l}\right\}$ as a judgment that the pairwise dis-similarities involving this stimulus are larger than the dis-similarity of the two non-outliers, i.e., that $D\left(x_{k},x_{j}\right)>D\left(x_{k},x_{l}\right)$ and also that $D\left(x_{l},x_{j}\right)>D\left(x_{l},x_{k}\right)$. Each trial thus contributes to estimates of choice probabilities for two triads, $\left(x_{k};x_{j},x_{l}\right)$ and $\left(x_{l};x_{j},x_{k}\right)$, and these judgments are tallied across the experiment. Note though that, in contrast to the “textures” and “faces” datasets, here the specific triadic comparisons that enter into the tallies depend on the participant’s responses.

For the “brightness” dataset, stimuli were generated in Python 3.10 and the NumPy library. Stimuli were displayed on a calibrated 24-inch ViewPixx monitor (1920×1080 pixel resolution, mean luminance 70 cd/m2, Vpixx Technologies, Inc.), running custom Python libraries that handle high bit-depth grayscale images (https://github.com/computational-psychology/hrl). Monitor calibration was accomplished using a Minolta LS-100 photometer (Konica Minolta, Tokyo, Japan). The display was viewed binocularly from a distance of 76 cm. The visual angle of the display was 39 degrees; each stimulus subtended 5 degrees, with the central disk subtending 1.67deg. The three stimuli were arranged in a triangular manner, 4 degrees equidistant from the center (Fig. 5A). There were 16 unique stimuli, consisting of all pairings of 8 values for the luminance of the center disk (14,33,55,78,104,131,163 and $\left.197\;cd/m^{2}\right)$ and 2 values of luminance for the surrounding annulus (0.77 and 226 cd/m^2^). The 16 stimuli generated $\left(\begin{array}{c} 16\\ 3 \end{array}\right)=560$ possible triplet combinations, which were presented in randomized order and position, constituting one block. Each session consisted of two blocks, and each participant ran four sessions. In total, we collected 4480 trials per participant. As each trial gives information for two triadic judgments (as mentioned above), there were 8960 triadic judgments per participant.

The “texture” and “faces” experiments were performed at Weill Cornell Medical College, in four participants (3F, 1M), ranging in age from 23 to 62. Participants MC and SAW (an author) were experienced observers and familiar with the “texture” stimulus set from previous studies; participants BL and ZK were naïve observers. All participated in the “textures” experiment; 2F (SAW and MC) participated in the “faces” experiment and neither had prior familiarity with those stimuli. The “brightness” dataset was performed at Technische Universität Berlin in three participants (1F,2M), ranging in age from 31 to 39. Participant JP was a naïve observer; participants GA (an author) and JXV were experienced observers. All participants had normal or corrected-to-normal vision. They provided informed consent following institutional guidelines and the Declaration of Helsinki, according to a protocol that was approved by the relevant institution.

In addition to the calculations described above, we also calculated the indices $I_{sym},I_{umi}$, and $I_{\text{addree}}^{'}$ for surrogate datasets. Surrogate datasets were constructed two ways. The “flip all” surrogate was created by randomly selecting triplets and flipping all choice probabilities (replacing $R_{obs}(r;x,y)$ by $\left.1-R_{obs}(r;x,y)\right)$ for the three triads within the selected triplets. The “flip any” surrogate was created by randomly selecting individual triads, and flipping the choice probabilities for the selected triads. Since the indices are sums of values that are independently computed either from triplets or tents, the exact means and standard deviations of the surrogate indices could be computed efficiently by exhaustive sampling of each triplet or tent separately, rather than approximated via a random sampling. Note also that the “flip all” surrogate leaves $I_{sym}$ unchanged, since the two criteria for symmetric dis-similarities (eqs. (2.3) and (2.4)) simply swap when all choice probabilities are flipped. The maximum-likelihood parameters a and h (eq. (3.4)) for the surrogates are also identical to those for the original datasets, since the prior is unchanged by flipping the choice probabilities.

Finally, we estimated the standard errors for the indices calculated from the original datasets via a jackknife on triplets (for $I_{sym},I_{umi}$) or tents (for $I_{\text{addrree}}^{'}$). Maximum-likelihood parameters a and h were not re-calculated for the jackknife subsets, as pilot analyses confirmed that removal of one triplet or tent made very little change in the maximum-likelihood value.

### Results

#### Textures

**Figure 2.**
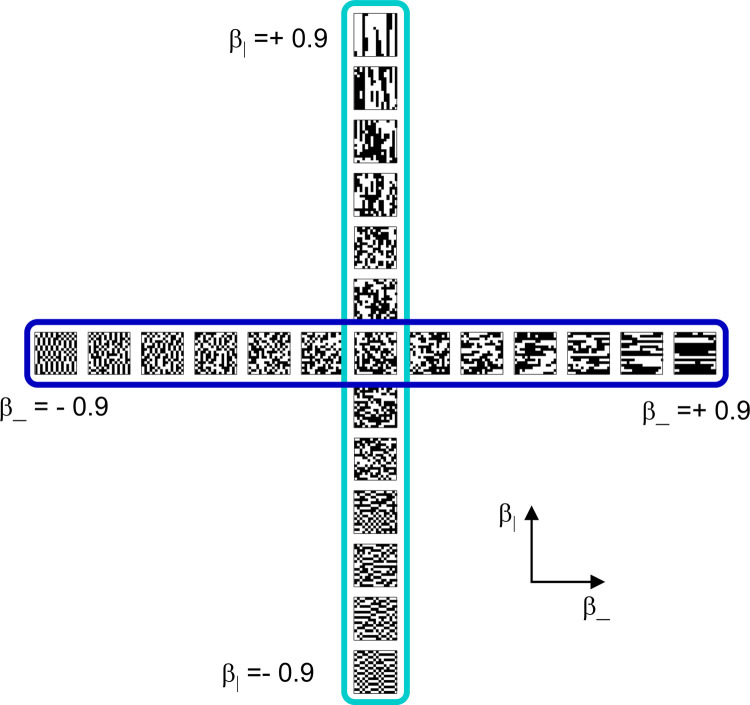

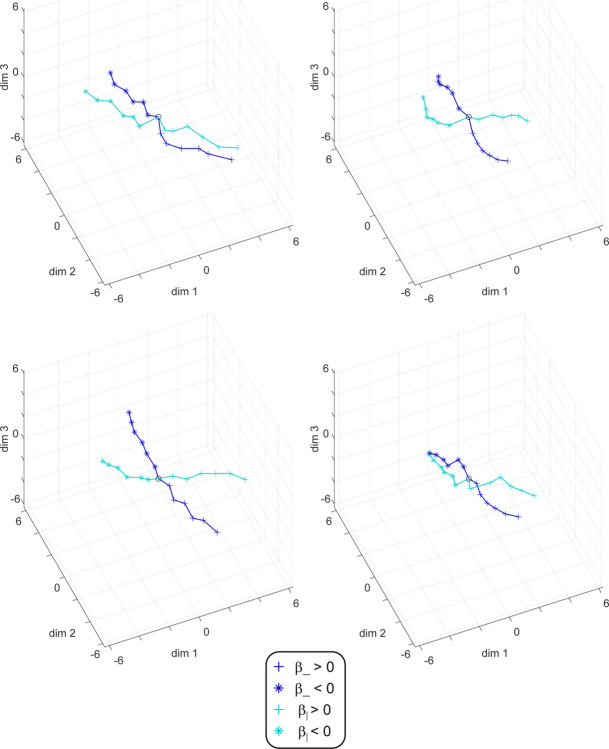
Panel A: Stimuli used in the texture experiment. Each stimulus is an array of 16 × 16 black or white checks. For stimuli enclosed in dark blue, checks are correlated (or anticorrelated) along rows. Correlation strength is parameterized by $\beta_{-}$, where $\beta_{-}>0$ indicates positive correlation and $\beta_{-}<0$ indicates negative correlation. For stimuli enclosed in light blue, checks are correlated (or anticorrelated) along columns, similarly parameterized by $\beta_{|}$. The full stimulus set consists of 6 equally-spaced values positive and negative values of $\beta_{-}$ and $\beta_{|}$, and an uncorrelated stimulus (center), where $\beta \_=\beta {=}_{|}0$. Panel B: Multidimensional scaling of similarity judgments for the stimuli in panel A for four participants. The data from each participant have been rotated into a consensus alignment via the Procrustes procedure (without rescaling). Lines connect stimuli along each of the rays in Panel A. One unit indicates one just-noticeable difference in an additive noise model (Waraich & Victor, 2022).

The “textures” experiment made use of the stimulus space described in (Victor & Conte, 2012), a 10-dimensional space of binary textures with well-characterized discrimination thresholds (Victor, Thengone, Rizvi, & Conte, 2015). We chose a two-parameter component of this domain (Figure 2A) that allowed a focus on testing for compatibility for addtree structure. The two parameters chosen, $\beta_{-}$ and $\beta_{|}$, determine the nearest-neighbor correlations in the horizontal or vertical direction: the probability that a pair of adjacent checks have the same luminance (either both black or both white) is (1+β)/2, and the probability that a pair of adjacent checks have the opposite luminance (one black, the other white) is (1-β)/2. Other than these constraints, the textures are maximum-entropy (see (Victor & Conte, 2012) for details). For these experiments, we chose values of $\beta_{-}$ or $\beta_{|}$ from −0.9 to 0.9 in steps of 0.15. That is, six stimuli had positive values of $\beta_{-}(0.15,0.30,0.45,0.60,0.75,0.90)$ with $\beta_{1}=0$, six had the corresponding negative values of $\beta_{-}$, six had positive values of $\beta_{|}$ with $\beta_{-}=0$, six had negative values of $\beta_{|}$ with $\beta_{-}=0$, and one had $\beta_{-}=\beta_{|}=0$. In the experiment, each stimulus example was unique– that is, a stimulus is specified by a particular $\left(\beta ,\beta_{|}\right)$ pair, but the texture example used on each trial was a different random sample from that texture.

The rationale for this stimulus set is that we anticipated that certain subsets of stimuli would be more compatible with the addtree model than others. The basis for these expectations is shown in Figure 2B, which presents non-metric multidimensional scaling of the similarity data. This analysis, carried out with the procedure detailed in (Waraich & Victor, 2022), uses a maximum-likelihood criterion to place the 25 stimulus points in a space, so that the Euclidean distances between them best account for the choice probabilities (assuming a uniform, additive decision noise). Consistently across participants, the points along each stimulus axis ($\beta_{-}$ or $\beta_{|}$) map to a gradually curving trajectory. For this reason, we anticipate that comparison data from the stimuli on one of these trajectories (the 13 points with either $\beta_{-}$ or $\beta_{|}$ equal to zero, here called an “‘axis”) when analyzed in isolation, will be close to an addtree model. However, the two trajectories are not perpendicular: rays with same signs of β meet at an acute angle of 〖45° or less. That is, stimuli with strong positive correlations $\left(\beta_{-}>0\right.$ compared to $\beta_{1}>0$) are seen as relatively similar to each other. This is anticipated to make the subset consisting of the 13 points with either $\beta_{-}$ or β positive (a “vee”) inconsistent with the addtree model, as the shortest perceptual path between two points at the end of the positive $\beta_{-}$ or $\beta_{|}$ rays is much shorter than a path that traverses each ray back to the origin. Similar reasoning indicates that the vee formed by the two negative rays should also be inconsistent with an addtree model. Note, though, that this intuition assumes that the Euclidean distances in Figure 2B are an accurate account of the perceptual dis-similarities; the analysis via $I_{\text{addtree}}^{'}$ does not make this assumption.

**Figure 3.**
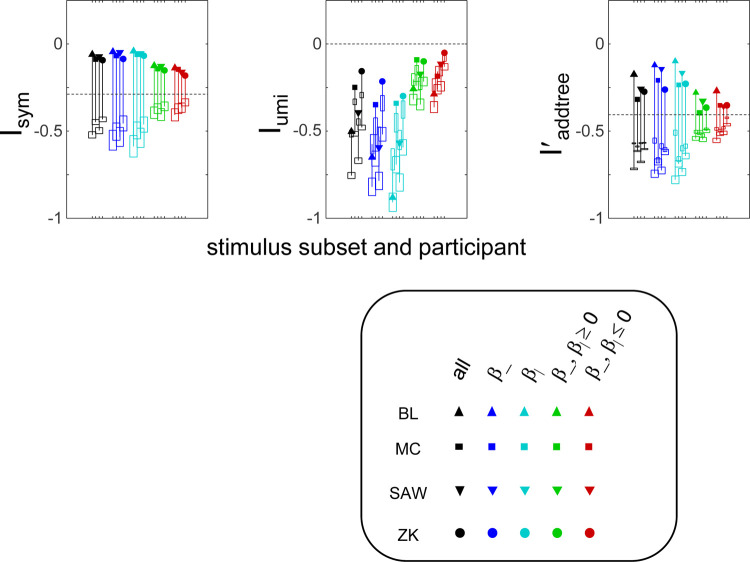
Indices $I_{sym},I_{umi}$, and $I_{\text{addree}}^{'}$ for the similarity judgments in the texture experiment. Stimulus subsets are indicated by symbol color; participants by symbol shape. The vertical extent of the wide boxes indicate pm 1 standard deviation for the “flip any” surrogates; the vertical extent of the narrow boxes indicate pm 1 standard deviation for the “flip all” surrogates (not plotted for $I_{umi}$, as this index is unchanged by the “flip all” operation). The thin vertical lines are to aid visualization, and do not represent ranges. Standard errors for the experimental datasets are smaller than the symbol sizes. Null-hypothesis values of the indices are indicated by the horizontal dashed line.

Figure 3 shows the indices $I_{sym},I_{umi}$, and $I_{\text{addrree}}^{'}$ computed from the full datasets for each participant, and for the axis and vee subsets. As expected from the above analysis, the addtree index $I_{\text{addree}}^{'}$ is substantially higher for the “axis” subsets than for the “vee” subsets, and has an intermediate value for the full dataset. Note that “axis” and “vee” subsets are in terms of the number of stimuli, and were collected simultaneously within a single experiment. This finding supports the efficacy of $I_{\text{addree}}^{'}$ in providing a characterization of the dataset regarding consistency with the addtree model. In all cases, it is higher than the *a priori* value, and substantially higher than values computed from surrogate datasets in which choice probabilities are randomly flipped. This latter point indicates (not surprisingly) that for all of these subsets, there are portions of the data that are more consistent with an addtree model than chance.

For this dataset, values of $I_{sym}$ were quite close to zero (usually >−0.1), indicating that nearly all $\left(>e^{-0.1}\right)$ of the posterior distribution of choice probabilities was consistent with a symmetric dissimilarity. $I_{umi}$, which measures consistency with the ultrametric model, was typically −0.25 or less, substantially below the *a priori* value of zero. But interestingly, the highest values of $I_{umi}$ were seen in the “vee” subsets, suggesting a partially hierarchical structure -e.g., that the two directions of correlation formed categories. As was the case for $I_{\text{addrree}}^{'}$, all indices were higher than for surrogates constructed by randomly flipping the choice probabilities. For $I_{sym}$, this is unsurprising, as randomly flipping choice probabilities would be unlikely to lead to a set of symmetric judgments. For $I_{umi}$, this finding indicates that, even though the ultrametric model is excluded, the data has islands of consistency with the ultrametric structure.

The above results were insensitive to the parameters a and h of the prior for the distribution of choice probabilities (eqs. (2.7) and (3.4)). The Dirichlet parameter a obtained by maximum likelihood (eq. (2.10)) ranged from 0.25 to 1.25 (with the lowest values for the full texture dataset), but very similar results as Figure 3 were obtained with setting a=0.5 for all datasets. For $I_{umi}$, the limit in (3.15) was estimated by setting h=0.001 but similar values were obtained for h=0.01. The findings for $I_{sym}$ and $I_{\text{addree}}^{'}$, here shown for h=0, were not substantially changed when h was determined by maximum likelihood. These values of h were typically quite small (median, 7 × 10^−5)^).

#### Faces

The “faces” experiment used stimuli drawn from the public-domain library of faces, at https://faces.mpdl.mpg.de/imejil, which contained color photographs of 171 individuals, stratified in three age ranges (“young”, “middle”, “old”). We randomly selected two males and two females from each age range, and for each individual in the faces database, used the two example photographs with neutral expressions, for a total of 24 unique images (2 genders × 3 age ranges × 2 individuals × 2 photographs of each).

**Figure 4.**
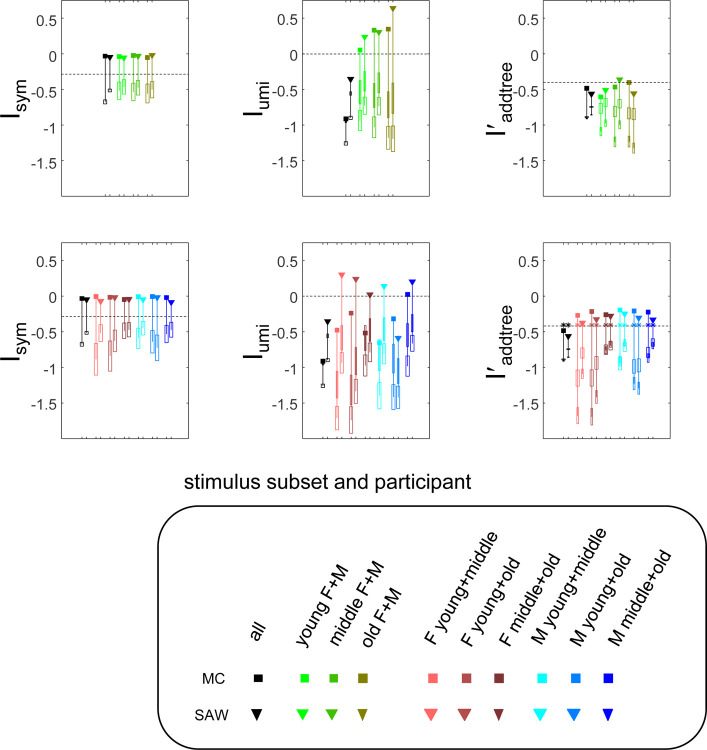
Indices $I_{sym},I_{umi}$, and $I_{\text{addrree}}^{'}$ for the similarity judgments in the faces experiment. Stimulus subsets are indicated by symbol color; participants by symbol shape. Upper row: full stimulus set (black symbols), and subsets partitioned by age. Lower row: full stimulus set (black symbols, repeated), subsets partitioned by gender, with two age ranges each. Other graphical conventions as in Figure 3.

The rationale for this choice of stimuli was that the above hierarchical organization might lead to a similarity structure close to ultrametric behavior. As shown in Figure 4, upper row, while this was not the case for analysis of the full dataset ($I_{umi}<0$, the *a priori* level), it was the case for the 8-stimulus subsets within each age bracket $\left(I_{umi}>0\right)$. Values of $I_{umi}>0$ were also seen for some subsets subdivided by gender (restricted to two age ranges, to equate the number of stimuli), as shown in Figure 4, lower row. Values of $I_{\text{sym}}$ were again quite close to zero (usually > −0.1), indicating strong consistency with a symmetric dis-similarity. Values of $I_{\text{addree}}^{'}$ were similar to the a priori value, but much larger than for the surrogates. As was the case for the texture experiment, these results were insensitive to the parameters a and h of the prior for the distribution of choice probabilities. Here, values of the Dirichlet parameter a obtained by maximum likelihood ranged from approximately 0.1 to 0.5; results similar to those of Figure 4 were obtained with setting a=0.3 for all datasets. Also as was the case for the texture experiment, findings for $I_{\text{sym}}$ and $I_{\text{addtree}}^{'}$, were not substantially changed when h was determined by maximum likelihood – even though the typical values of h were larger (median, 6 × 10^−2^), supporting the idea that many underlying choice probabilities were close to 0.5.

#### Brightness

**Figure 5.**
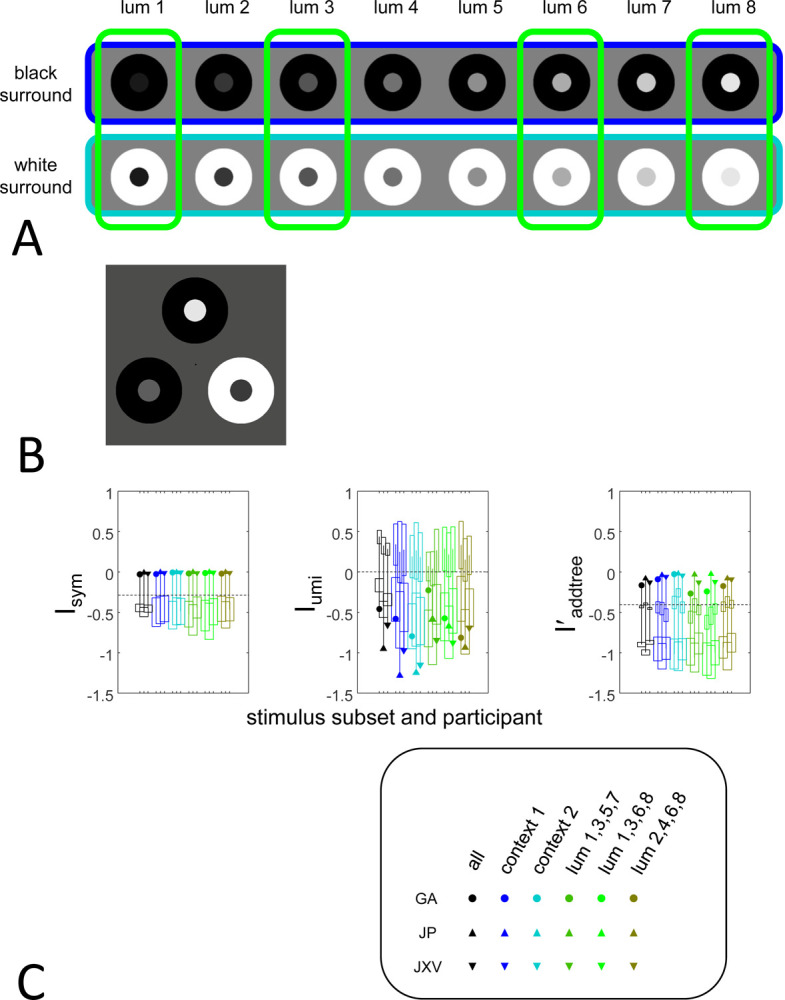
Panel A: Stimuli for the brightness experiment. Each stimulus had a disk-and-annulus configuration, in which the disk had one of 8 luminances (columns) and either a black (upper row) or a white (lower row) surround annulus. The colored lines encircle three of the stimulus subsets used in Panel C. Panel B: A sample trial. Panel C: Indices $I_{sym},I_{umi}$, and $I_{\text{addrree}}^{'}$ for similarity judgments in the brightness experiment for the full stimulus set (black symbols), 8-element subsets with only one of the two kinds of surround (blue symbols), and 8-element subsets with both surrounds (green symbols). Other graphical conventions as in Figure 3.

The “brightness” experiment consisted of judgments of brightness dis-similarity for the set of disk-and-annulus stimuli as shown in Figure 5A. This disk-and-annulus configuration has been extensively used to study the effect of the context surround on the appearance of the inner disk (Gilchrist, 2006; Wallach, 1948). A light surround is expected to have make the inner disk appear darker, and conversely, darker surround is expected to make the inner disk appear lighter. While it is generally assumed that this shift in appearance is along a one-dimensional brightness continuum, the evidence is ambiguous(Murray, 2021). For example, Madigan and Brainard (Madigan & Brainard, 2014) found that one dimension suffices to explain brightness similarity ratings, while Logvinenko & Maloney (Logvinenko & Maloney, 2006) found that dissimilarity ratings under different illuminations required a 2-dimensional perceptual space.

This open question motivated the stimuli used in the present experiment: a gamut of 8 disk luminances, presented with either of 2 surround contexts (Figure 5A). Participants judged the brightness of the inner disk for triplets constructed from all possible combinations of disk luminance and surround (Figure 5B). If brightness is one dimensional, then dis-similarity judgments for the full set of stimuli should be consistent with a one-dimensional model, which is a special case of an addtree model. If, on the other hand, the surround produces differences in appearance that are not one dimensional, then the full set of judgments should be inconsistent with addtree. Under this hypothesis, restricting the judgments to stimuli with the same surround (the subsets encircled by the dark and light blue lines inFigure 5A) should recover a one-dimensional structure and consistency with an addtree model, while restriction to a same-sized set but with two kinds of surrounds (green lines in Figure 5A) should remain inconsistent.

Figure 5C shows the results. For the full stimulus set (black symbols), $I_{\text{addree}}^{'}$ is close to zero (> 0.17), and substantially higher than the *a priori* value, for all three participants. Even higher indices are found for the 8-element stimulus subsets of only black $\left(I_{\text{addree}}^{'}>0.09\right)$ or only white $\left(I_{\text{addtree}}^{'}>0.05\right)$ surrounds (blue symbols in Figure 5C). This is consistent with the notion that, when context is held constant, dis-similarity judgments are highly consistent with a one-dimensional space. However, when $I_{\text{addrree}}^{'}$ was computed for 8-element subsets of the stimuli in which judgments were made across two surrounds (green symbols in Figure 5C), $I_{\text{addtree}}^{'}$ was lower, and varied substantially across participants. GA always the lowest value ($I_{\text{addtree}}^{'}-0.26$ to −0.17 ) and JP the highest value close to zero $\left(I_{\text{addree}}^{'}-0.08\right.$ to −0.03).

These findings show that when the surround context is constant, judgments are highly consistent with an addtree model, but there is inter-observer variability when judgments are made across two surround contexts. The variability is not surprising, as previous research has shown that individual idiosyncrasies can play a substantial role when disk-in-context stimuli are used to study brightness or color (Radonjic, Cottaris, & Brainard, 2015). Our method seems to be capturing these inter-individual differences, but – as we are focusing on a demonstration of the analysis methods – we do not attempt to probe the basis for this difference here.

Similar to the texture and faces experiments, values of $I_{sym}$ are all close to zero for the brightness dataset, indicating consistency with symmetric dis-similarity judgments. Ultrametric indices $I_{umi}$ are below the *a priori* value for all cases, indicating inconsistency with an ultrametric model, as expected for a one-dimensional continuum.

Also as in the texture and faces experiments, results were robust to changes of analysis details.Values of the Dirichlet parameter a obtained by maximum likelihood ranged from approximately 0.07 to 0.22; results similar to those of Figure 4 were obtained with setting a=0.1 for all datasets. Findings for $I_{\text{sym}}$ and $I_{\text{addtree}}^{'}$ were not substantially changed when h was determined by maximum likelihood, yielding values of h with a median of 5 × 10^−2^, comparable to the faces dataset.

## Discussion

The main contribution of the paper is to advance a strategy for connecting similarity judgments of a collection of stimuli to inferences about the structure of the domain from which the stimuli are drawn. The starting point is an experimental dataset in which the judgments are assumed to be independently drawn binary samplings distributed according to the underlying choice probabilities. We assume that for each triad (a reference stimulus and two comparison stimuli), the comparison stimulus that is more often judged to be more similar to the reference is at a shorter distance from it, but we do not assume, or attempt to infer, a relationship between choice probabilities and the distances. This approach also takes into account the possibility that each triad may have its own “local” transformation that links choice probability and distance. While we recognize that judgments may be uncertain, we refrain from postulating a noise model or a decision model – or even that sensory or decision noise is uniform throughout the space.

Despite the paucity of assumptions, we show that it is possible to characterize dis-similarity judgments along three lines: consistency with symmetry, consistency with an ultrametric model, and consistency with an additive tree model. These characteristics are functionally significant aspects of a domain’s organization. Symmetry (or its absence) has implications for the mechanism(s) by which comparisons are made (Tversky, 1977; Tversky & Gati, 1982). For symmetric similarity judgments, addtree models, but not ultrametric models, are consistent with the Tversky contrast model (Sattath & Tversky, 1977). More broadly, semantic domains are anticipated to be consistent with a hierarchical model of similarity judgments (ultrametric or addtree), while domains of features are not (Kemp & Tenenbaum, 2008; Saxe et al., 2019). It is also worth noting that one-dimensional domains are a special case of the addtree model, so the present approach can address whether the apparent “curvature” in a one-dimensional perceptual space can be eliminated by alternative choices of the linkage between distance and decision – a limitation of the analysis in (Bujack, Teti, Miller, Caffrey, & Turton, 2022). Furthermore, our method is sensitive enough to reveal inter-individual differences: for some participants data are consistent the addtree model and for others, not (or less so) – consistent with other studies of the influence of context (Radonjic et al., 2015), and an interesting area for further investigation.

### Comparisons to other methods

The present strategy, whose overriding consideration is to keep assumptions at a minimum, is complementary to other ways of analyzing similarity judgments. A classical and commonly-used approach, non-metric multidimensional scaling (de Leeuw & Heiser, 1982; Tsogo et al., 2000), explicitly postulates that the original data (here, the choice probabilities) reflect a monotonic transformation of a metric distance. The distance is taken to be the Euclidean distance, but distances in a hyperbolic or spherical geometry can also be used. An important related approach for one-dimensional models is maximum-likelihood difference scaling (Knoblauch & Maloney, 2008; Maloney & Yang, 2003), which– via a decision model – takes into account the noisy nature of psychophysical judgments. This approach can also be extended to multidimensional models, but the need for a decision model remains (Victor et al., 2017; Waraich & Victor, 2022).

Our approach also contrasts with that of topological data analysis via persistent homologies (Dabaghian et al., 2012; Giusti et al., 2015; Singh et al., 2008; Zhou et al., 2018). Like our approach, TDA avoids the need to postulate a specific relationship between dis-similarities and distances, as the Betti numbers are calculated from a sequence of graphs that depend only on the rank order of similarity judgments. However, construction of this sequence of graphs does require a globally uniform linkage between triadic judgments and relative distance, and also, that every pairwise distance is included in the measured triads. The characterizations yielded by TDA area also complementary: they focus on dimensionality and homology class, rather than the characterizations considered here.

Finally, we note another approach that directly seeks to identify features of ultrametric behavior in neural data. Treves (Treves, 1997) developed an information-theoretic measure of dis-similarity of neural responses to faces. The strategy for seeking evidence of ultrametric behavior was to examine the ratio, within each triplet, of the middle distance to the largest. This ratio, which would be 1 for an ultrametric, was found in that study to be closer to 1 than for expected by chance. Nonparametric generalizations of this approach may permit a relaxation of the assumed linkage between extensions the information-theoretic measure and dis-similarity, and even an evaluation of addtree models – but in contrast to our approach, it begins with a set of responses to each stimulus, rather than a sampling of triadic comparisons.

### Caveats

Keeping assumptions to a minimum necessarily leads to certain limitations. The indices for symmetry and addtree structure reflect necessary, but not sufficient, conditions. Moreover, these indices do not measure the goodness of fit of a model, and are not directly applicable to model comparisons: the indices merely measure to what extent the data act to concentrate the *a priori* distribution of choice probabilities into the subset of choice probabilities that have a particular characteristic. Thus, it is important to recognize that the extent of concentration will depend on the typical coverage of each triad: a greater number of trials of each triad will data provides better estimates of the underlying choice probabilities, and thus, can move the indices further from their *a priori* values. For this reason, the examples above (axes vs. vees in Figure 3; subdivision by age vs. gender in Figure 4, same context vs. different contexts in Figure 5C) focus on comparisons of indices between datasets with a similar number of stimuli, and a similar coverage of each.

### Extensions and open issues

There are open questions that are raised by the present approach, as well as some directions in which it might be extended.

At a practical level, if the present analysis indicates that a dataset is consistent with an ultrametric or addtree model, to what extent can the ultrametric or addtree structure be determined? Existing methods for taking this step require a complete set of dis-similarity measures (Abraham, Bartal, & Neiman, 2015; Barthélemy & Guénoche, 1991; Hornik, 2005; Sattath & Tversky, 1977), along with the assumption that the transformation from triadic choice probabilities into dis-similarities is uniform across the space. Choice probabilities provide constraints even without this assumption – for example certain relationships among the triadic judgments involving four points are sufficient for a local addtree model – but it is unclear whether these constraints are sufficient for a global model, or how such a global model can be determined.

The observation that a metric that obeys the four-point condition can always be realized by the path metric on a weighted acyclic graph (Buneman, 1974) suggests the possibility of a succession of further characterizations of a set of choice probabilities. By definition, acyclic graphs have no three-cycles. An isolated 3-cycle with nodes $a_{1},a_{2}$, and $a_{3}$ can always be removed by adding a node c, with distances $d\left(c,a_{1}\right)=\left(d\left(a_{1},a_{2}\right)+d\left(a_{1},a_{3}\right)-d\left(a_{2},a_{3}\right)\right)/2$, etc.; this quantity is guaranteed to be non-negative via the triangle inequality, and $d\left(c,a_{1}\right)+d\left(c,a_{2}\right)=d\left(a_{1},a_{2}\right)$. Thus, ruling out the four-point condition on distances via the condition (4.5) implies that the dis-similarity structure cannot be realized on a weighted acyclic graph, or on a weighted graph with only isolated three-cycles. Consequently, a graph with two non-disjoint three-cycles or a four-cycle, is required. Similarly, more elaborate conditions analogous to (4.5) then rule out realizing the dis-similarities on graphs with less than some specified level of cycle structure. For example, if the conditions (4.5) hold for two quadruplets with three points in common, then the four-cycles required by each configuration must have three points in common, so a five-cycle, or two linked four-cycles, or multiple edge-sharing three-cycles, are required to be present in a graph that accounts for the dis-similarities among the five points.

In this regard, it is interesting to note that the ultrametric condition and the four-point condition have a similar structure: both state that of three numbers (three single distances for the ultrametric, three pairwise sums for the four-point condition), the largest two must be identical. This intriguing similarity raises the further possibility of a sequence of analogous conditions, each specifying a progressively less-restrictive aspect of a set of dis-similarity judgments – such as compatibility with planar graphs, or statements about the Betti numbers.

## Figures and Tables

**Figure 6.**
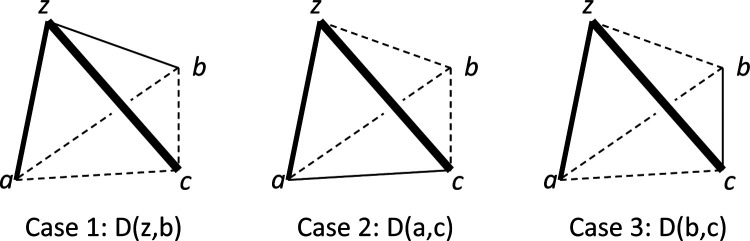
Three rank-orderings of the dis-similarities among four points consistent with falsification of the conjunction (4.5). In all cases, D(z,c) is largest (heavy line) and D(z,a) is second-largest (intermediate line). The third-largest dis-similarity can be either D(z,b),D(a,c), and D(b,c) (thin solid lines). See Appendix 3 for details.

## Appendix 1: Metric spaces

This appendix demonstrates that consistency of a set of choice probabilities with a symmetric dis-similarity implies consistency with a metric-space structure.

A metric space is defined to be a set of points {x,y,z,…}, along with a distance d(x,y) that associates each pair of points, and that satisfies three properties:

(6.1)
d(x,y)≥0 and d(x,y)=0⇔x=y,

(6.2)
symmetry: d(x,y)=d(y,x),

and

(6.3)
the triangle inequality: d(x,y)≤d(x,z)+d(y,z).

As we have generically assumed (in eq. (1.7)) that dis-similarity D(x,y) satisfies (6.1) and now add the assumption that it is symmetric, we need to show that we can replace D(x,y) – which need not satisfy the triangle inequality – by a distance d(x,y) which both satisfies the triangle inequality and accounts for the choice probabilities via

(6.4)
R(r;x,y)>1/2⇔d(r,x)<d(r,y).

Since our central assumption is that the rank-order of dis-similarities accounts for whether a choice probability is less than or greater than $\frac{1}{2}$ (eq. (1.5), which is (6.4) with D instead of), eq. (6.4) will hold when the distance d is any monotonic transformation of the dis-similarity *D*:

(6.5)
d(x,y)=F(D(x,y)).

So it suffices to find a monotonic transformation for which d(x,y) satisfies the triangle inequality.

A suitable transformation for this purpose is

(6.6)
$$F\left(u\right)=\{\begin{array}{c} 0,u=0\\ 1+\frac{u}{1+u},u>0 \end{array}.$$

For distinct elements a and b,D(a,b)>0 and consequently d(a,b)=F(D(a,b)) is between 1 and 2.

So the left-hand side of (6.3)is always < 2 and each term on the right-hand side is > 1, and the triangle inequality holds.

## Appendix 2: Addtree Models and the Four-Point Condition

This Appendix demonstrates some well-known and basic (Buneman, 1974; Dobson, 1974; Sattath & Tversky, 1977) relationships between the four-point condition, the triangle inequality, and the ultrametric inequality.

To see that the four-point condition (4.1) implies the triangle inequality, we set w=x. Then (4.1) becomes

(7.1)
$$\text{None of the three quantities}\left\{\begin{array}{c} d\left(u,v\right)\\ d\left(u,x\right)+d\left(v,x\right)\\ d\left(u,x\right)+d\left(v,x\right) \end{array}\right\}\text{is strictly greater than the other two}.$$

That is, d(u,v)≤d(u,x)+d(v,x), which is the triangle inequality.

To see that the ultrametric inequality (3.1) implies the four-point condition, we first relabel the points u,v,w, and x so that d(u,v) is the smallest distance, and

(7.2)
d(u,v)≤d(u,w)≤d(u,x).

Applied to the triangle with vertices u,v, and w, the ultrametric inequality -- which says that all triangles are isosceles, with the two equal sides no shorter than the third -- yields,

(7.3)
d(u,v)≤d(u,w)⇒d(v,w)=d(u,w).

Similarly, applied to the triangle with vertices u,v, and x, the ultrametric inequality yields,

(7.4)
d(u,v)≤d(u,x)⇒d(v,x)=d(u,x).

Combining these two yields

(7.5)
d(u,w)+d(v,x)=d(v,w)+d(u,x).

Applied to the triangle with vertices u,w, and x, the ultrametric inequality yields,

(7.6)
d(u,w)≤d(u,x)⇒d(w,x)=d(u,x).

Combining this with (8.2) yields

(7.7)
d(u,v)+d(w,x)=d(u,v)+d(u,x)≤d(v,w)+d(u,x).

Together, (8.5) and (8.7) constitute the four-point condition.

### Examples

For four distinct points on a line in the order and distances given by the ordinary Euclidean distance d(y,z)=|z-y|, the addtree conditions hold. Taking u<v<w<x,

(7.8)
d(u,w)+d(v,x)=(w−u)+(x−v)=(w−v)+(x−u)=d(u,x)+d(v,w),

while

(7.9)
d(u,v)+d(w,x)=(v−u)+(x−w)=(v+x)−(u+w)<(w+x)−(u+v)=(x−u)+(w−v)=d(u,x)+d(v,w),

However, the ultrametric inequality does not in general hold, since d(u,v)<d(u,w)<d(u,x).

For the standard distance between four distinct in general position in a plane, the four-point condition does not hold, since the three pairsums are typically distinct.

## Appendix 3: Local Sufficiency for the Four-Point Condition

Here, we show that falsification of the conjunction (4.5) suffices to ensure that the dis-similarities are consistent with a local addtree model. More precisely, we show that if, for each relabeling of a set of four points, (4.5) does not hold, then there is a monotonic transformation d(x,y)=F(D(x,y)) of the dis-similarities into distances, for which the four-point condition (4.1) holds. We will always choose F to be of the form

(8.1)
$$F\left(D\right)=\{\begin{array}{c} D+k,k\geq D_{0}\\ D,D<D_{0} \end{array}.$$

so the demonstration rests on finding an appropriate choice of $D_{0}$ and k We note that we can ignore whether the quantity that results from the monotonic transformation satisfies the triangle inequality, since Appendix 2 shows that the four-point condition implies the triangle inequality.

We refer to the three quantities in the four-point condition (4.1) and the corresponding sums of pairs of dis-similarities as “pairsums.” We at first assume that all dis-similarities are unequal, and consider the case of equality below.

Given that the dis-similarities are unequal, we relabel the points so that D(z,c) is the greatest dis-similarity. If D(z,c)+D(a,b) is not the largest pairsum, we choose $D_{0}=D(z,c)$ in (9.1). With this choice, d(z,c)+d(a,b)=D(z,c)+D(a,b)+k, but the other pairsums are unchanged. Setting k equal to the difference between D(z,c)+D(a,b) and the next-largest pairsum yields a transformation to distances that satisfies the four-point condition.

So we only need to consider cases where D(z,c) is the largest dis-similarity and D(z,c)+D(a,b) is the largest pairsum. In each case, we take $D_{0}>D(a,b)$ but smaller than the next-largest disparity. This increases D(z,c)+D(a,b) by k. We show that this transformation increases at least one of the other two pairsums by at least 2k, enabling one of the other two pairsums to “catch up” to D(z,c)+D(a,b). To do this, we need to show that for at least one of the other two pairsums, both terms are greater than D(a,b).

Given that D(z,c) is the largest dis-similarity, the second-largest dis-similarity cannot be D(a,b), since then (4.5) would hold. So the second-largest dis-similarity must share a point with the largest dis-similarity, and we can label the points so that this second-largest dis-similarity is D(z,a). D(a,b) cannot be the third-largest dis-similarity, since then all of (4.5) would hold. Thus, we may assume that D(z,c) is the largest dis-similarity, D(z,a) is the second-largest, and we consider the three possibilities for the third-largest dis-similarity, D(z,b),D(a,c), and D(b,c), in turn (Figure 6).

If the third-largest dis-similarity is (z,b) : Either D(a,c)>D(a,b) or D(b,c)>D(a,b), since otherwise (4.5) would hold. Thus, both terms of at least one of D(z,b)+D(a,c) or D(z,a)+D(b,c) are greater than D(a,b).

If the third-largest dis-similarity is D(a,c):D(a,b) cannot be larger than both D(z,b) and D(b,c), because then, a conjunction like (4.5) would hold with b at the vertex (we would have D(a,b) the largest of the tent at b, and D(z,c) the largest of the base.) So at least one of D(z,b)>D(a,b) or D(b,c)>D(a,b). Thus, both terms of either pairsum D(z,b)+D(a,c) or D(z,a)+D(b,c) are greater than D(a,b).

We now extend the above argument to the case in which the conjunction (4.5) is false after allowing some of the dis-similarities to be equal (but these equalities involve at most one of the two components of a pairsum, as specified following eq. (4.5)). We need to show that under these circumstances, there is still a monotonic distortion of the dis-similarities into distances for which the four-point condition holds. Assume otherwise – i.e., that there is a “rogue” configuration of dis-similarities Q that does not allow for such a monotonic distortion. If there is such a rogue configuration Q, we can find, for sufficiently small ε, another configuration $Q_{\varepsilon }$ with unique dis-similarities, whose dis-similarities within ε of Q at the ties, and equal to the dis-similarities in Q at the non-ties. The above construction yields a monotonic distortion $d_{\varepsilon }(u,v)=F_{\varepsilon }\left(D_{\varepsilon }(u,v)\right)$ that converts the dis-similarities in $Q_{\varepsilon }$ to distances that satisfy the four-point condition.

Now apply the limit of these monotonic transformations, ${lim}_{\varepsilon \to 0}\;F_{\varepsilon }$ to the dis-similarities in Q. (This limit must exist, and the convergence is uniform: if, two configurations $Q_{1}$ and $Q_{2}$ have dis-similarities that match to within an amount q, then the monotonic functions needed for $Q_{1}$ and $Q_{2}$ will differ by at most q). In this limit, the four-point conditions must hold, since they are simple linear functions of the distances, that hold for all sufficiently small ε>0. That F is monotonic follows from the monotonicity of each $F_{\varepsilon }$, and the uniform convergence of $F_{\varepsilon }$ to F.
