# Supplementary material for: Ordinal Characterization of Similarity Judgments
Source: ArXiv. 2025 Aug 6:arXiv:2310.07543v5. Originally published 2023 Oct 11. Preprint. [Version 5] (PMC10593068)
Supplement: Supplement 1 [file NIHPP2310.07543v5-supplement-1.pdf]

## Supplementary Material

**Supplementary figures.** Supplement to main text Figures 5 - 11

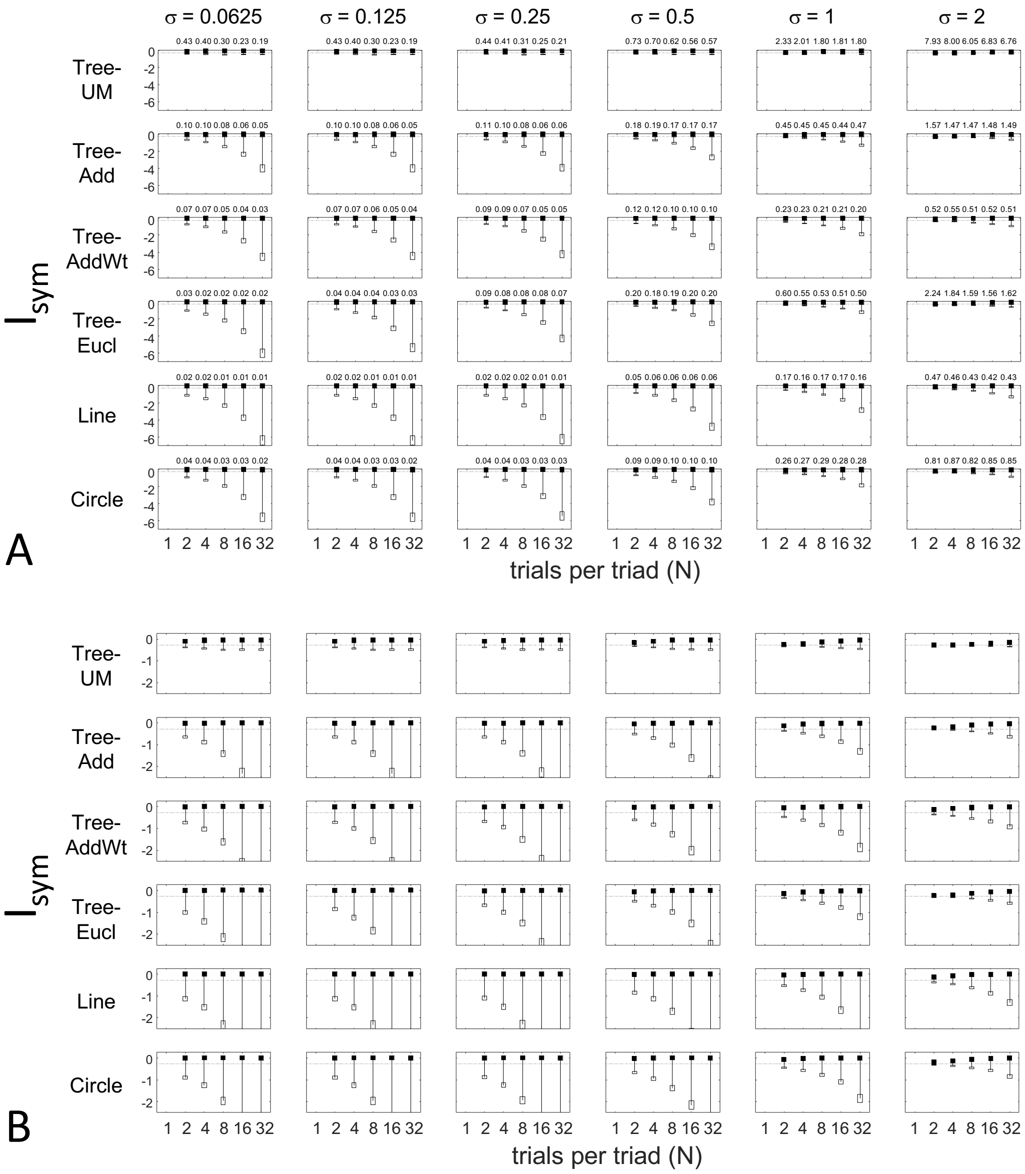


Figure S1: (supplement to main text Figure 5). Panel A. Behavior of $I_{\mathrm{sym}}$ for the 15-point configurations, a range of trials per triad ($N$, abscissa), and a range of decision rules ($\sigma$, standard deviation of added noise). Solid symbols indicate values of $I_{\mathrm{sym}}$ computed from simulated decisions. The dashed horizontal line indicates the *a priori* value for $I_{\mathrm{sym}}$. Hollow boxes indicate values of $I_{\mathrm{sym}}$ computed from "flip any" surrogates, showing the $\pm 1$ standard deviation range. The thin vertical lines are to aid visualization, and do not represent ranges. The beta-function prior (3.4) is used, with the value of the parameter $a$ (shown in fine print over each data point) determined by maximum likelihood. Panel B expands the vertical scale.

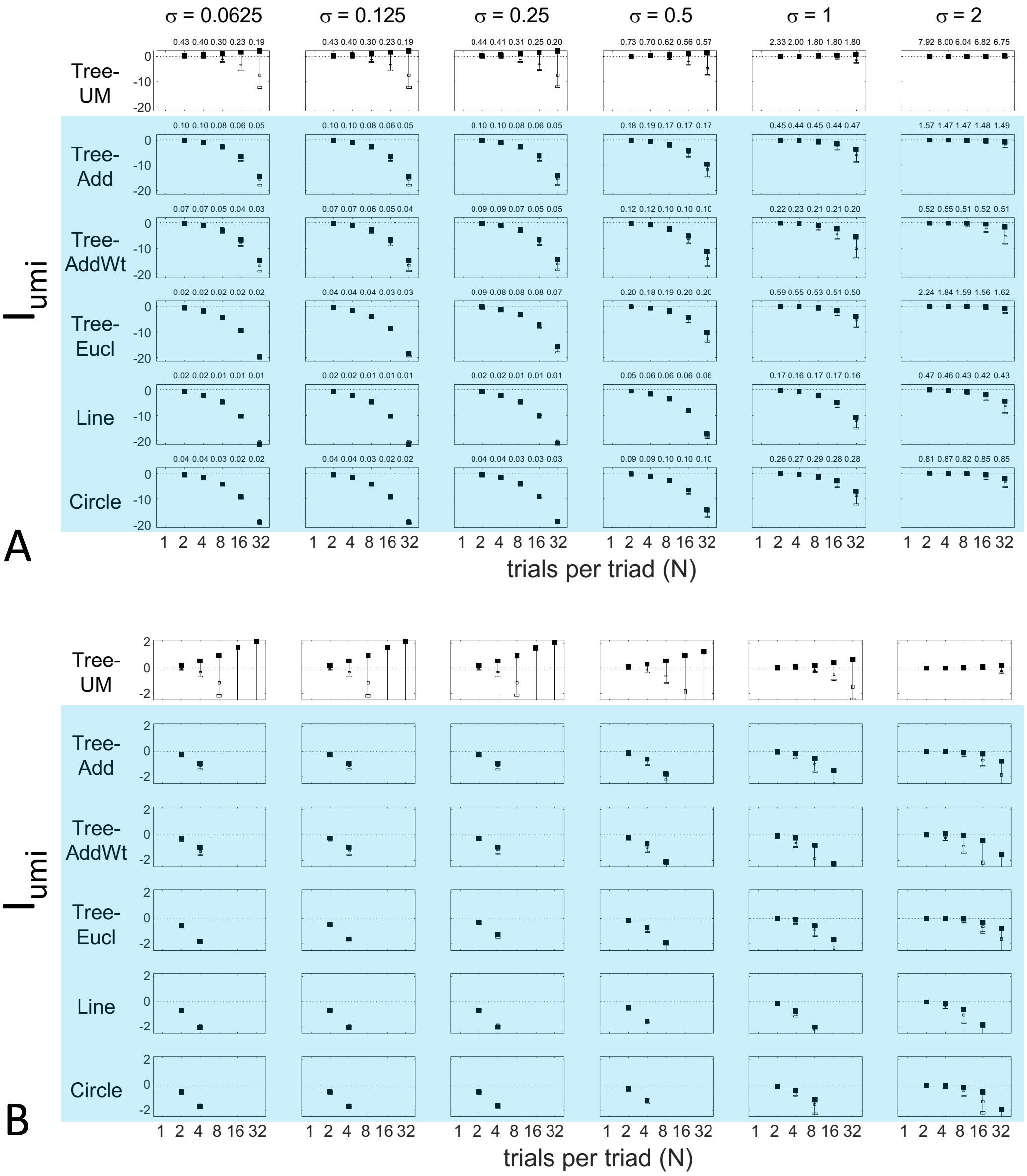


Figure S2: (supplement to main text Figure 6). Panel A. Behavior of $I_{\text{umi}}$ for the 15-point configurations, a range of trials per triad ($N$, abscissa), and a range of decision rules ($\sigma$, standard deviation of added noise). Solid symbols indicate values of $I_{\text{umi}}$ computed from simulated decisions. The dashed horizontal line indicates the *a priori* value for $I_{\text{umi}}$. Hollow boxes indicate values of $I_{\text{umi}}$ computed from surrogates, showing the $\pm 1$ standard deviation range: wider boxes for "flip any" surrogates, narrower boxes for "flip all" surrogates. The thin vertical lines are to aid visualization, and do not represent ranges. The modified prior (3.27) is used, with the value of the parameter $a$ (shown in fine print over each data point) determined by maximum likelihood and $h = 0.001$. Panel B expands the vertical scale. Blue overlay indicates the simulated datasets that are incompatible with the ultrametric property.

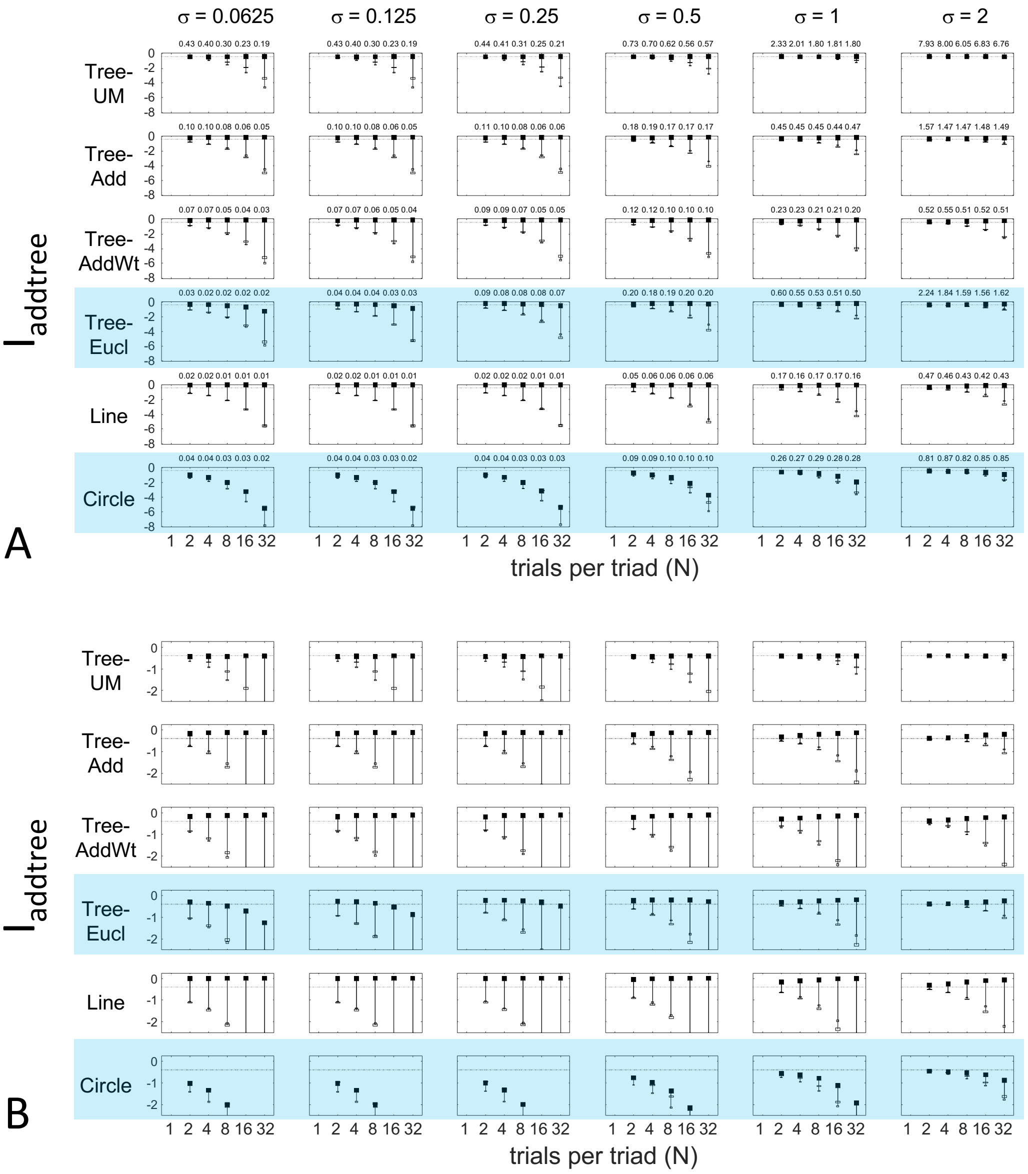


Figure S3: (supplement to main text Figure 7). Panel A. Behavior of $I_{\text{addtree}}$ for the 15-point configurations, a range of trials per triad ($N$, abscissa), and a range of decision rules ($\sigma$, standard deviation of added noise). Solid symbols indicate values of $I_{\text{addtree}}$ computed from simulated decisions. The dashed horizontal line indicates the *a priori* value for $I_{\text{addtree}}$. Hollow boxes indicate values of $I_{\text{addtree}}$ computed from surrogates, showing the $\pm 1$ standard deviation range: wider boxes for "flip any" surrogates, narrower boxes for "flip all" surrogates. The thin vertical lines are to aid visualization, and do not represent ranges. The beta-function prior (3.4) is used, with the value of the parameter $a$ (shown in fine print over each data point) determined by maximum likelihood. Panel B expands the vertical scale. Blue overlay indicates the simulated datasets that are incompatible with the addtree property.

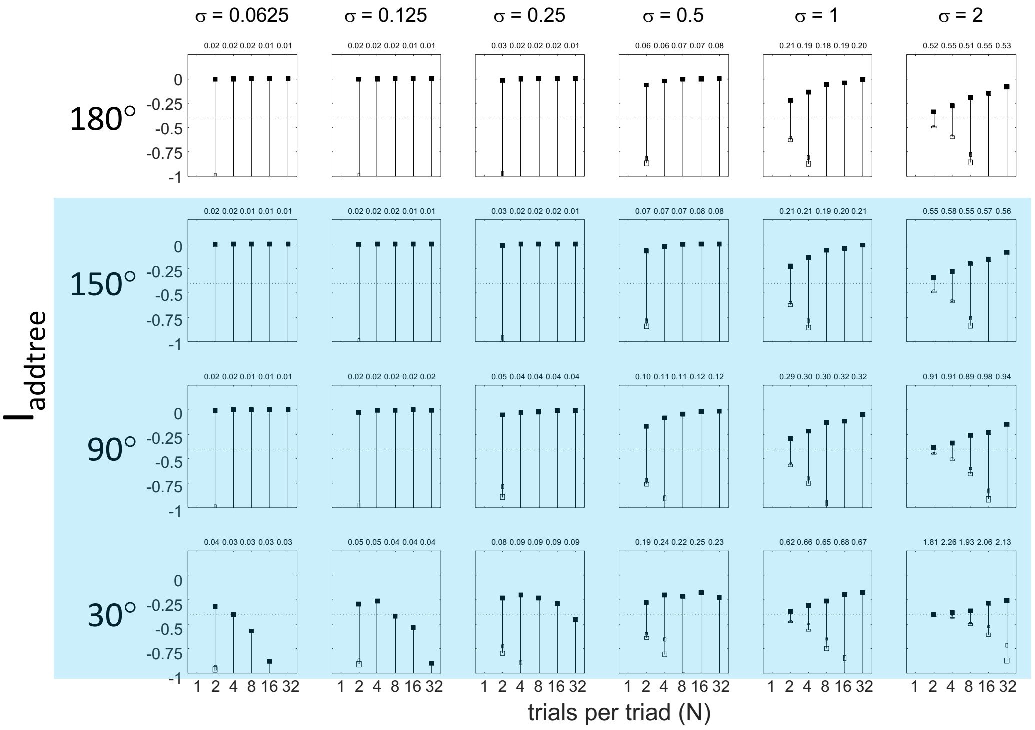


Figure S4: (supplement to main text Figure 8). Behavior of $I_{\text{addtree}}$ for the 13-point configurations, a range of trials per triad (abscissa), and a range of decision rules ($\sigma$, standard deviation of added noise). Blue overlay indicates the simulated datasets that are incompatible with the addtree property. Other graphical conventions as in Supplementary Figure S3.

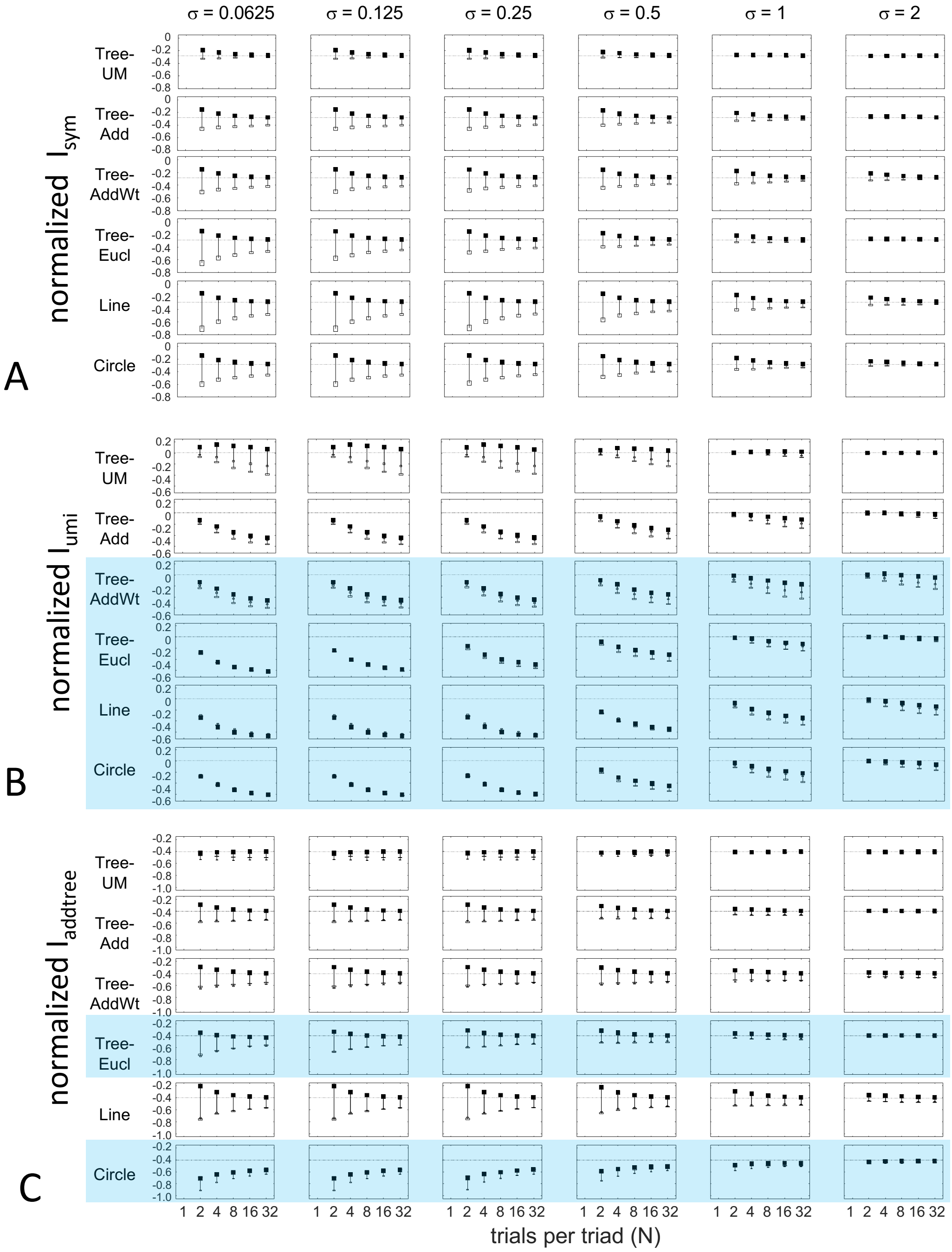


Figure S5: (supplement to main text Figure 9). Normalized values of $I_{\text{sym}}$ (panel A), $I_{\text{umi}}$ (panel B), and $I_{\text{addtree}}$ (panel C), for the 15-point configurations, a range of trials per triad ($N$, abscissa), and a range of decision rules ($\sigma$, standard deviation of added noise). Data from Supplementary Figures S1-S4, replotted after normalizing the deviation from the a priori value by the number of trials per triad: $I_0 + (I - I_0)/N$, where $I$ is the index from Supplementary Figures S1-S4, $I_0$ is the *a priori* value, and $N$ is the number of trials per triad. Blue overlay indicates the simulated datasets that are incompatible with the ultrametric property (B) or the addtree property (C). Other graphical conventions as in Supplementary Figures S1-S3.

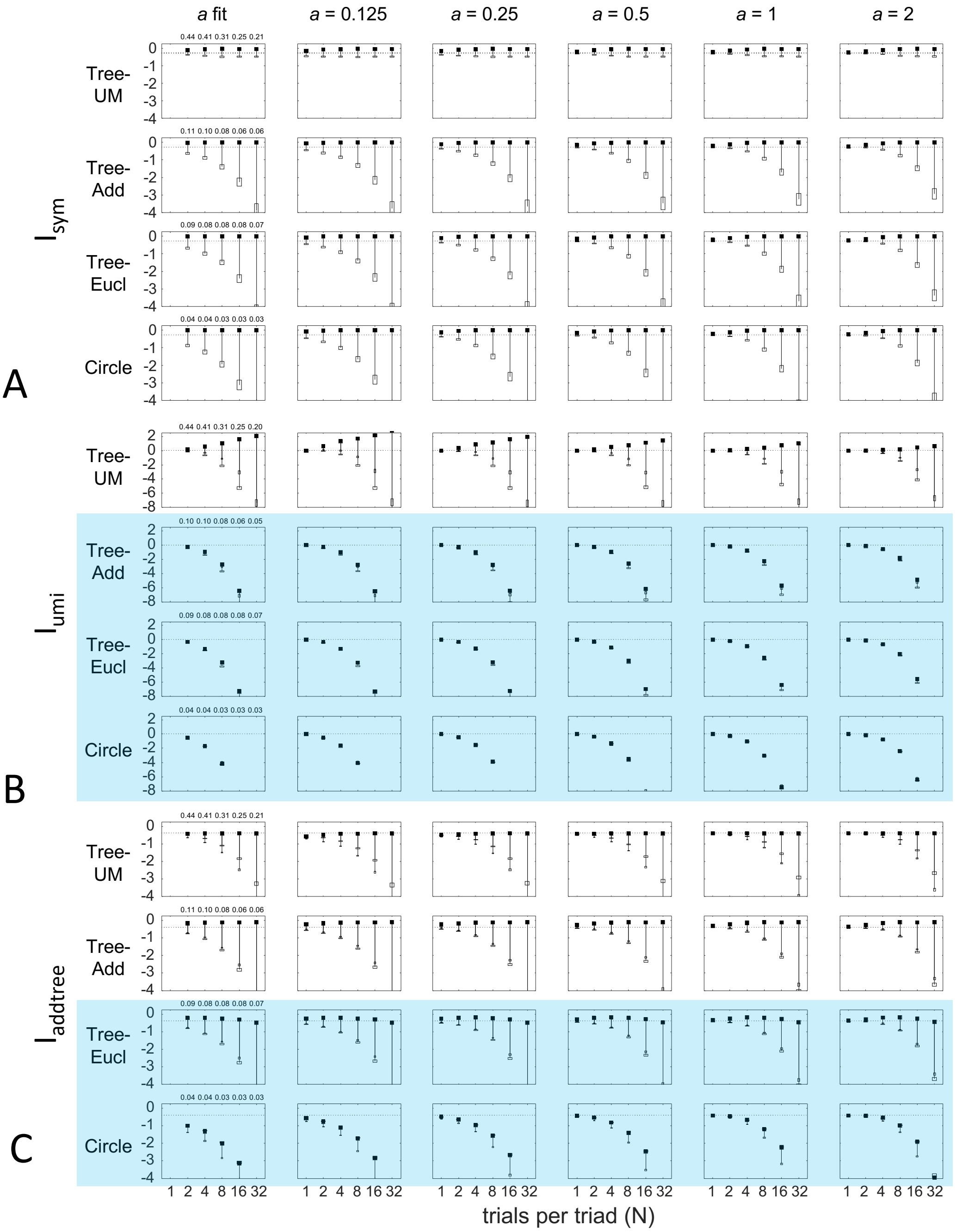


Figure S6: (supplement to main text Figure 10). Dependence on the prior's shape parameter, $a$, for $I_{\text{sym}}$ (panel A), $I_{\text{umi}}$ (panel B), and $I_{\text{addtree}}$ (panel C), for the 15-point configurations and a range of trials per triad ($N$, abscissa). The decision rule $\sigma = 0.25$ is used. Column 1: $a$ is determined by maximum likelihood; other columns: $a$ is assigned the value indicated over each column. The beta-function prior (3.4) is used for $I_{\text{sym}}$ and $I_{\text{addtree}}$; the modified prior (3.27) is used for $I_{\text{umi}}$ with $h = 0.001$. Blue overlay indicates the simulated datasets that are incompatible with the ultrametric property (B) or the addtree property (C). Other graphical conventions as in Supplementary Figures S1-S3.

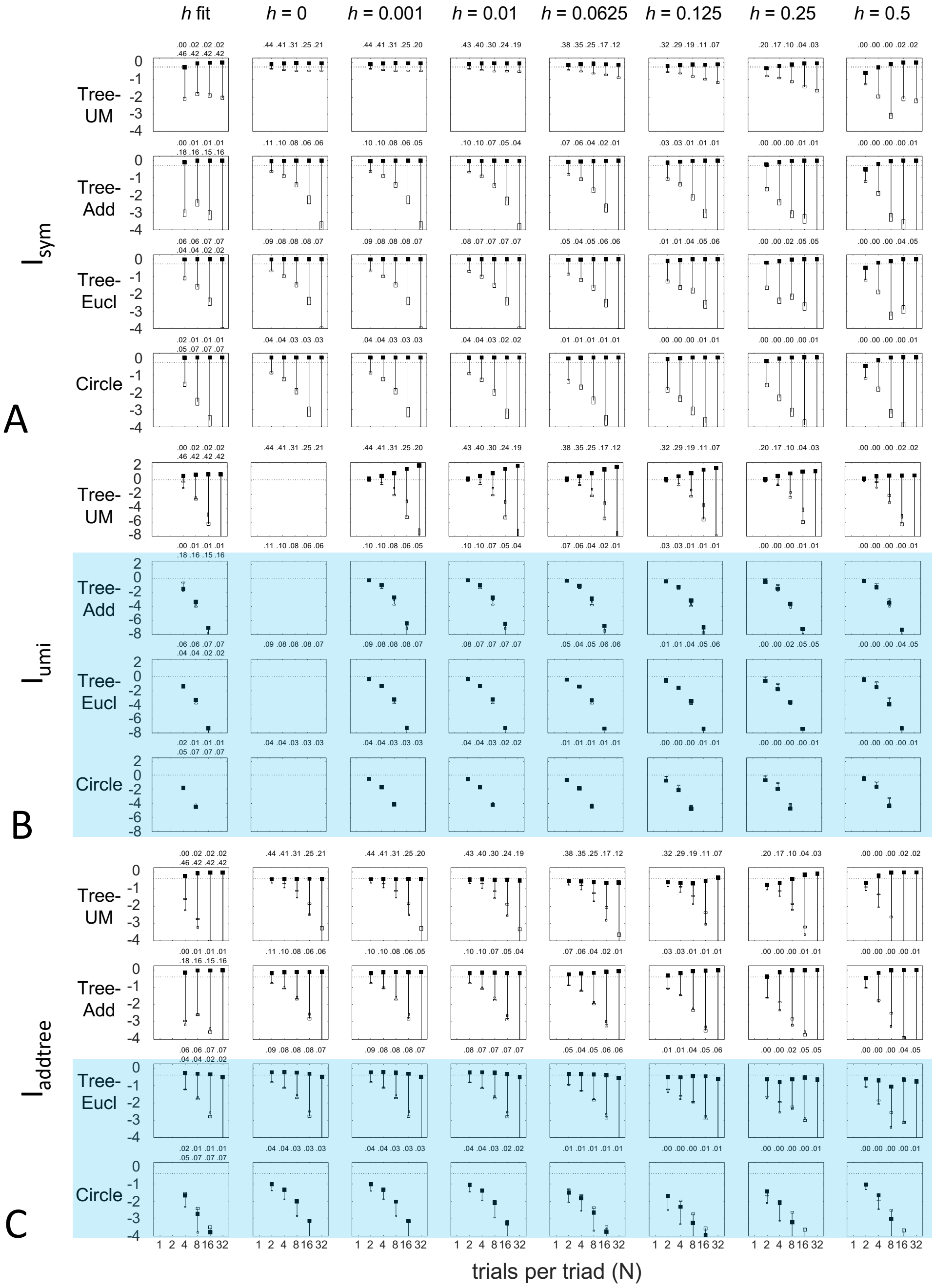


Figure S7: (supplement to main text Figure 11). Dependence on the prior's point mass parameter, $h$, for $I_{\text{sym}}$ (panel A), $I_{\text{umi}}$ (panel B), and $I_{\text{addtree}}$ (panel C), for the 15-point configurations and a range of trials per triad ($N$, abscissa). The decision rule $\sigma = 0.25$ is used. Column 1: $h$ is determined by maximum likelihood (shown in fine print in lower row over each data point); other columns: $h$ is assigned the value indicated over each column. The parameter $a$ (shown in fine print over each data point) is determined by maximum likelihood in all cases. The modified prior (3.27) is used for all indices. Note that $I_{\text{umi}}$ is undefined for $h = 0$ (panel B, second column). Blue overlay indicates the simulated datasets that are incompatible with the ultrametric property (B) or the addtree property (C). Other graphical conventions as in Supplementary Figures S1-S3.
