# Supplementary material for: Ordinal Characterization of Similarity Judgments
Source: ArXiv. 2025 Feb 12:arXiv:2310.07543v3. Originally published 2023 Oct 11. Preprint. [Version 3] (PMC10593068)
Supplement: Supplementary file 1 [file NIHPP2310.07543v3-supplement-1.pdf]

Supplementary Figures

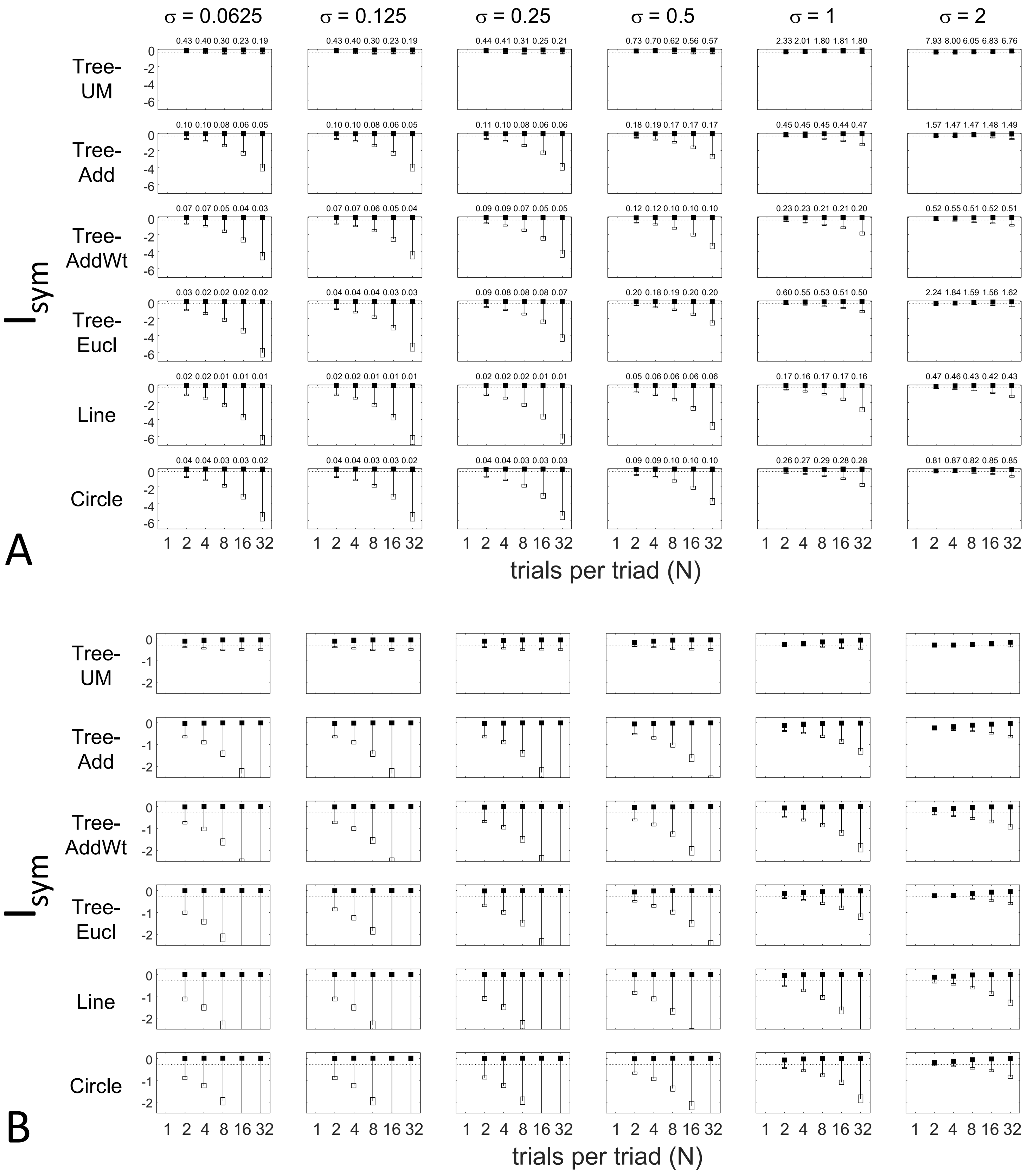

Supplementary Figure 1

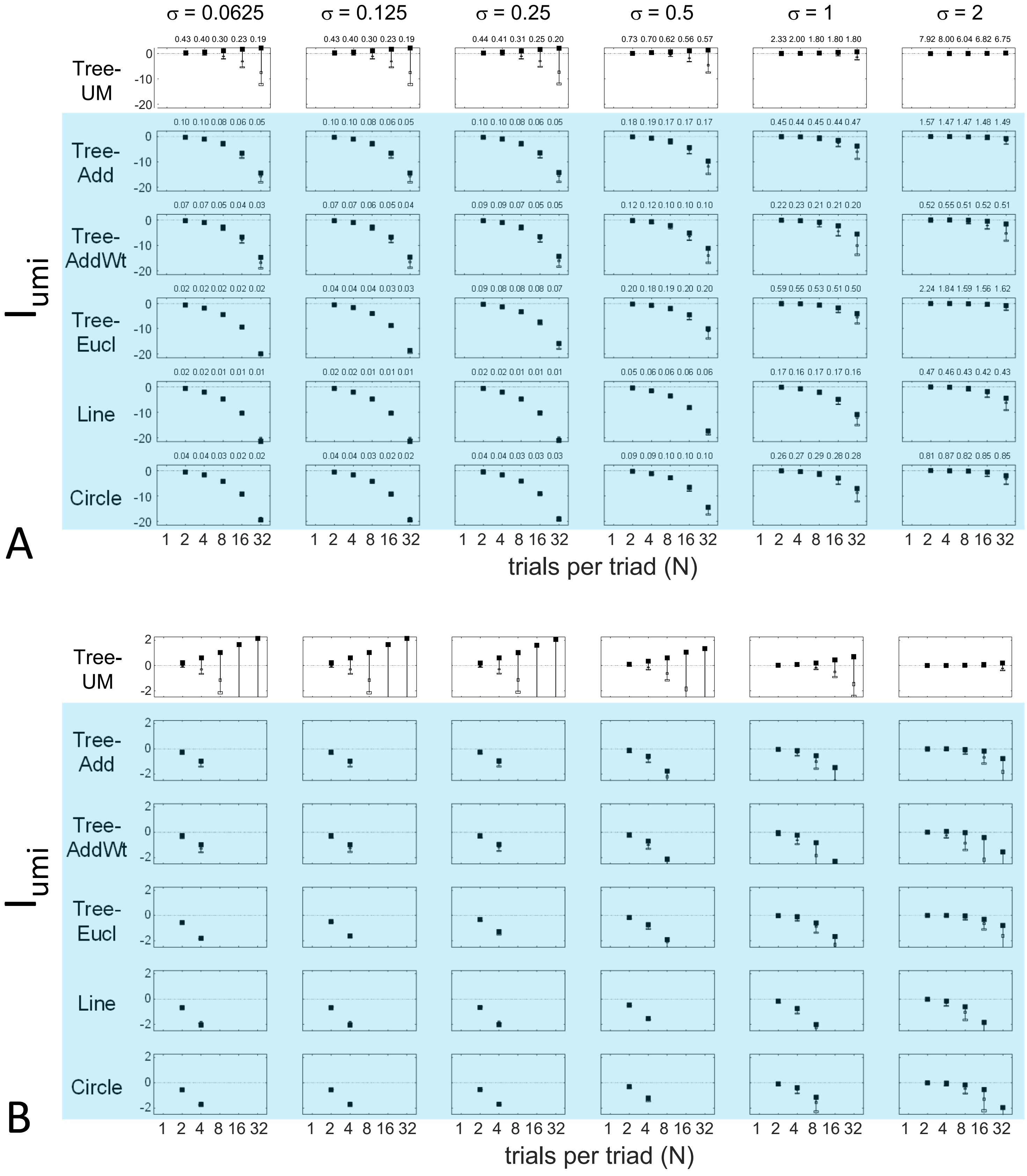

Supplementary Figure 2

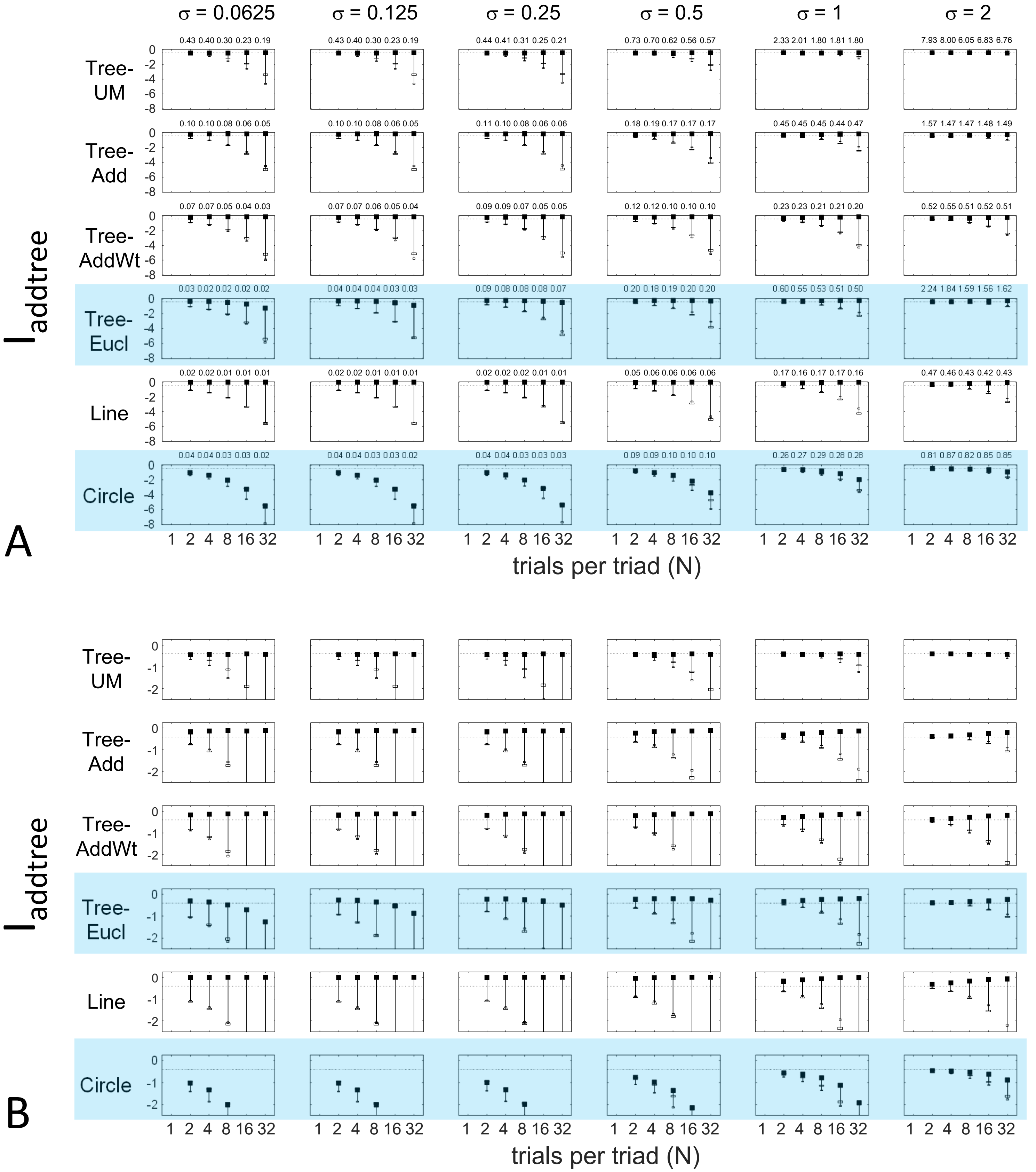

Supplementary Figure 3

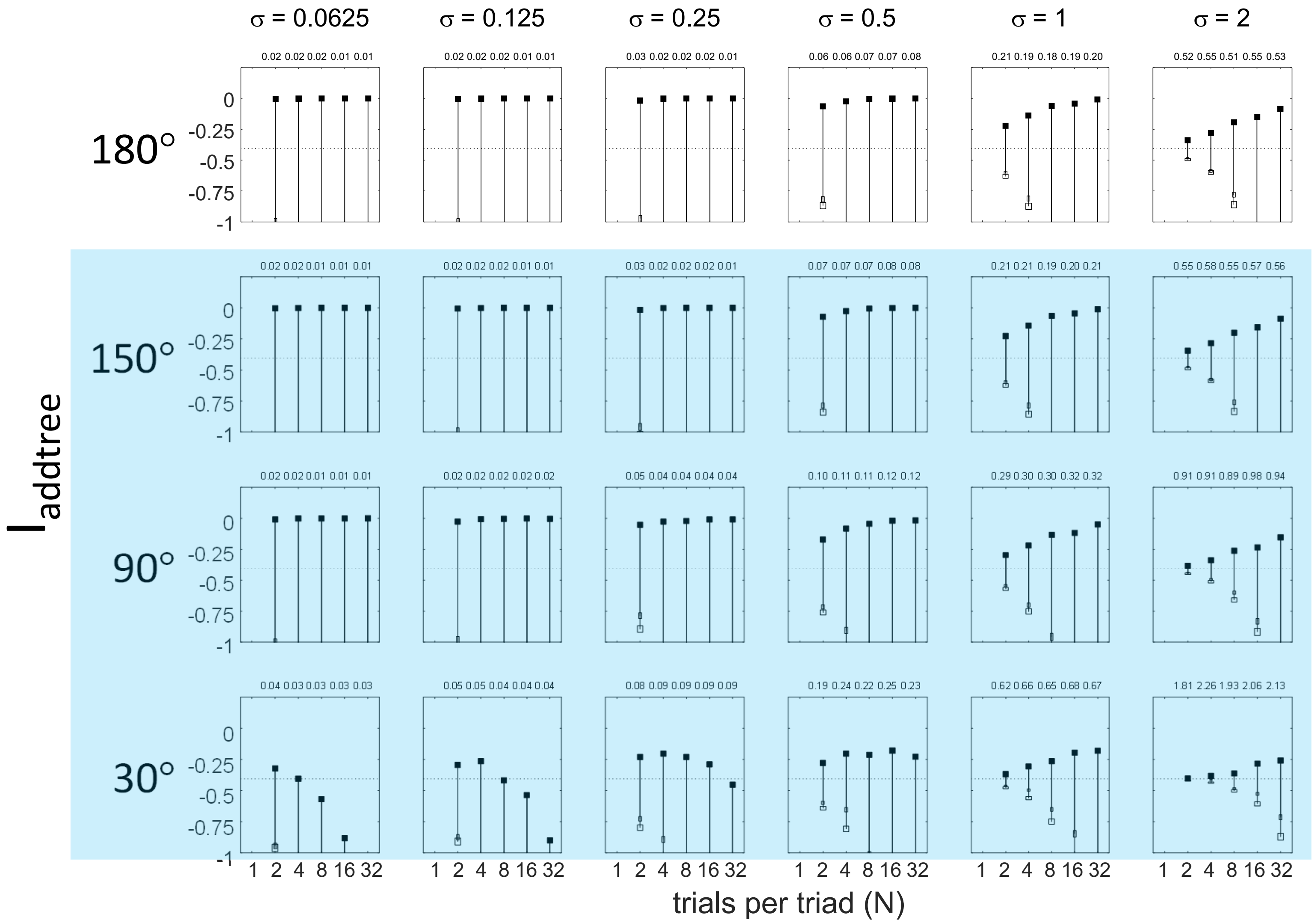

Supplementary Figure 4

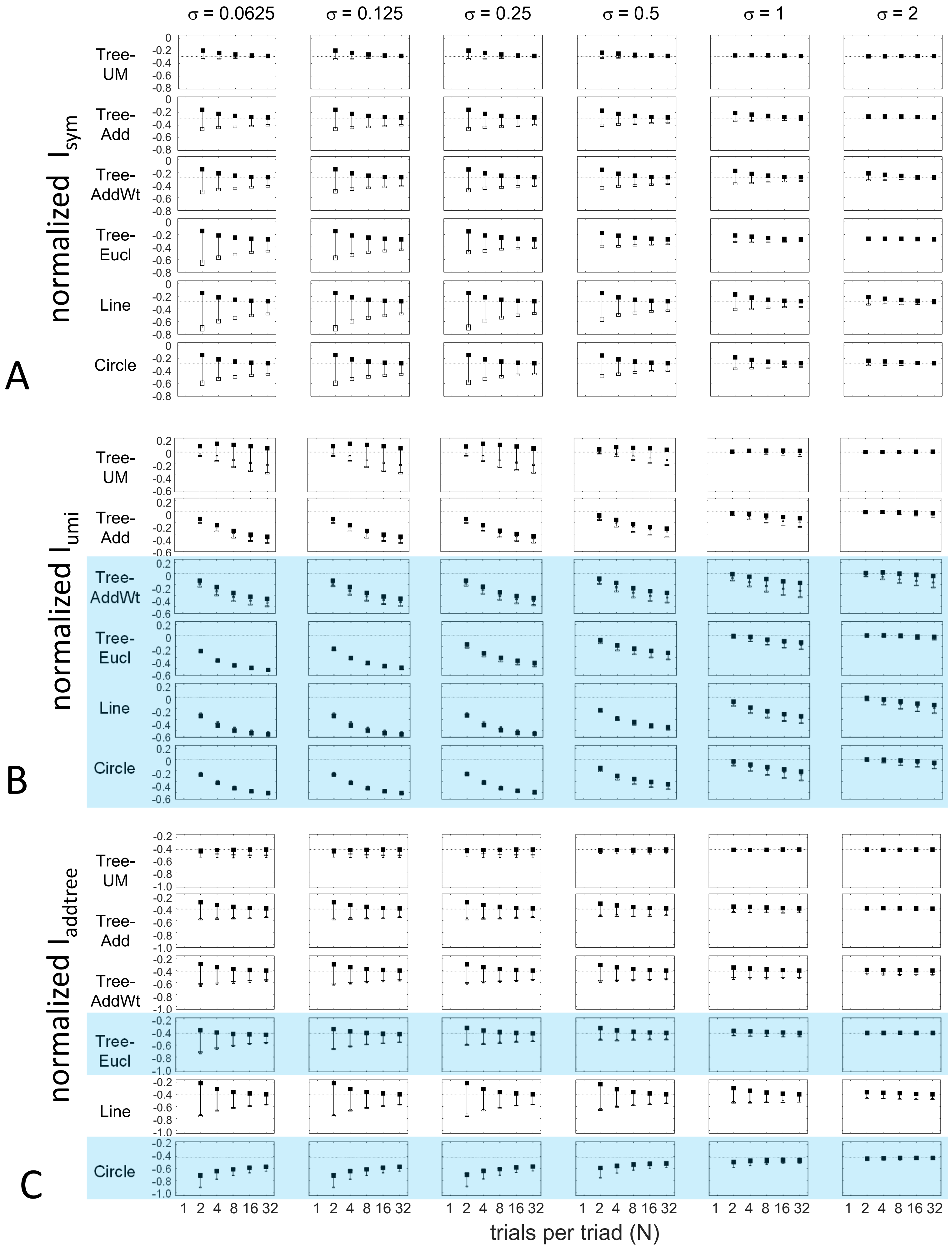

Supplementary Figure 5

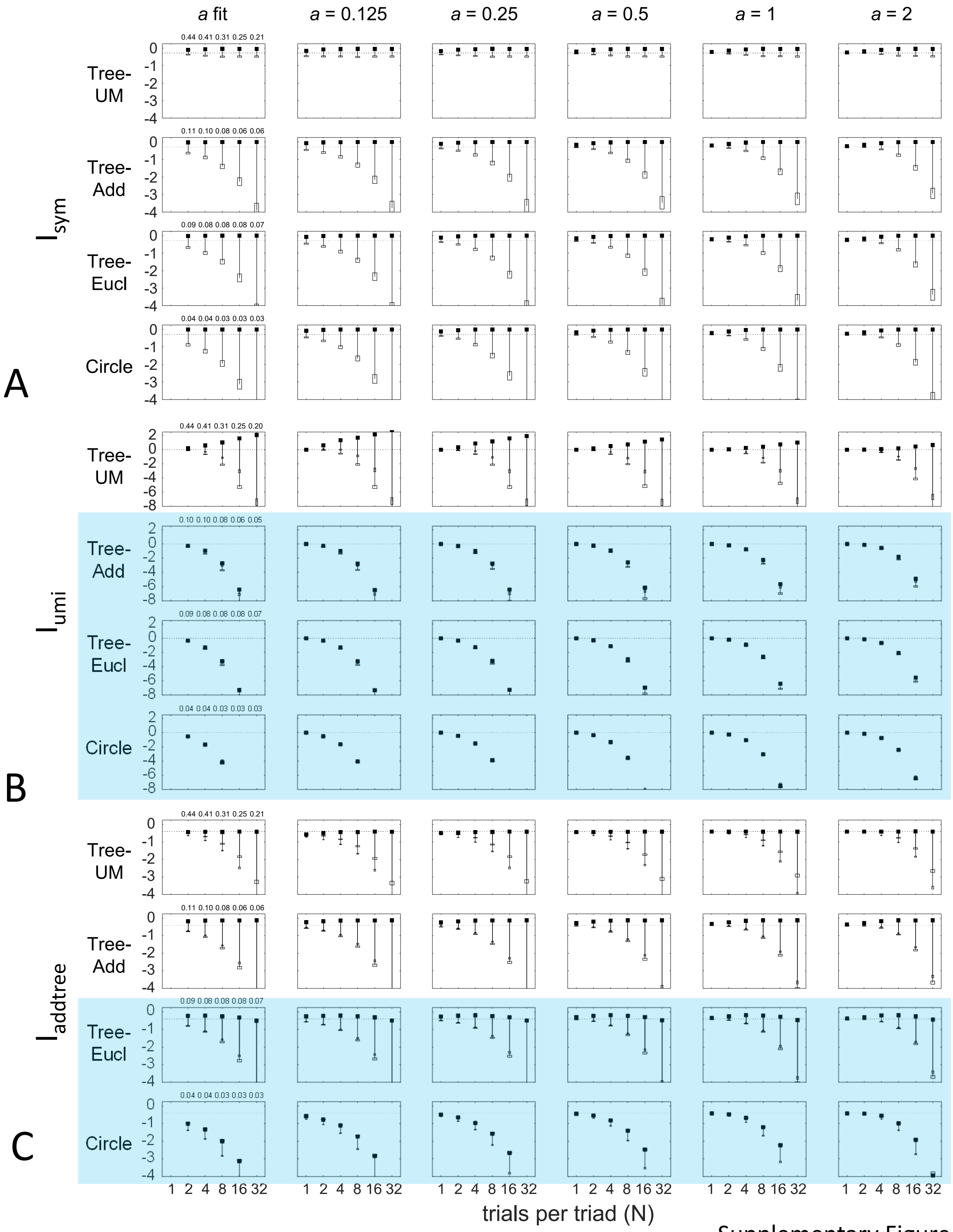

Supplementary Figure 6

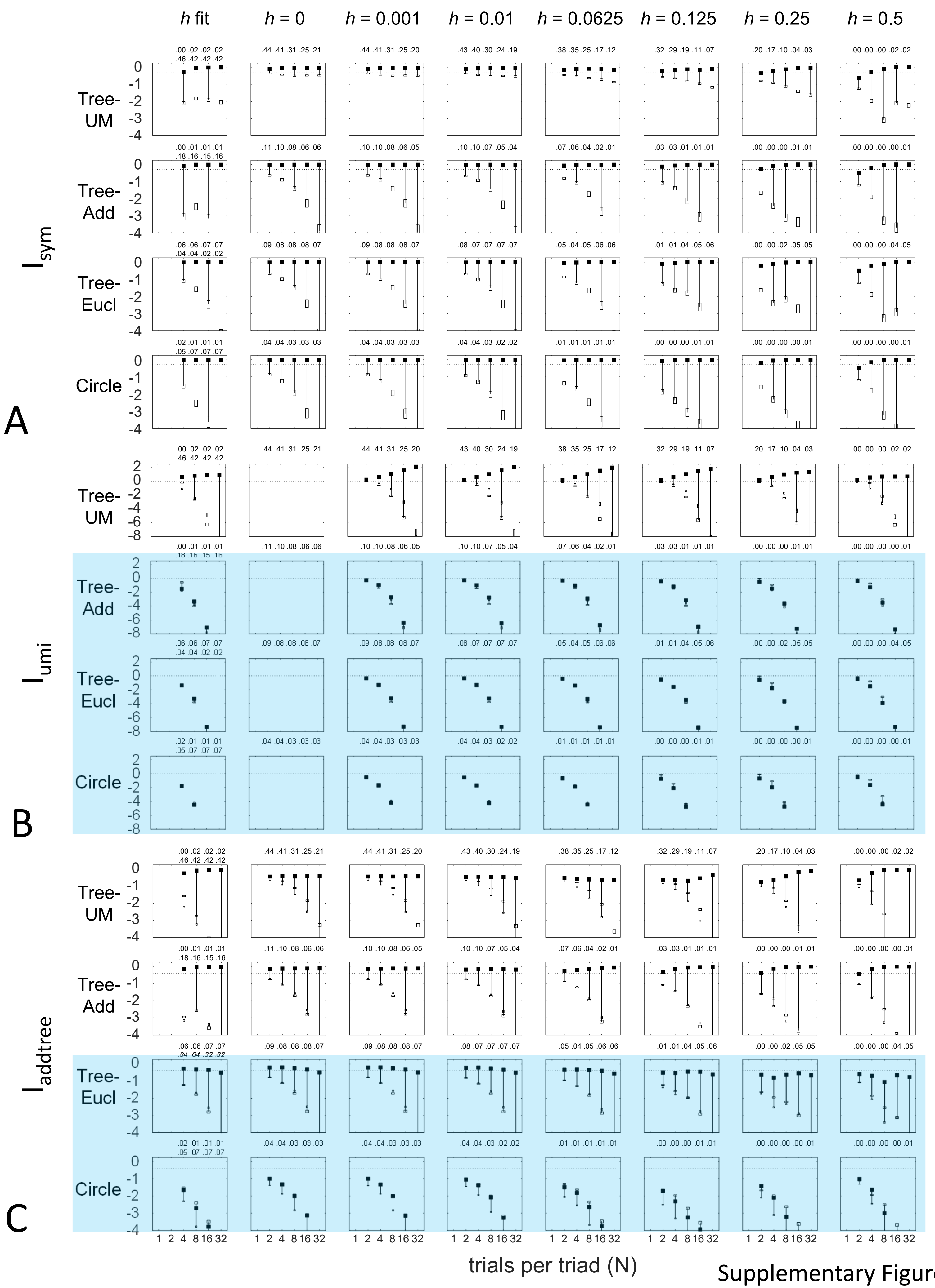

Supplementary Figure 7
