# Supplementary material for: Ordinal Characterization of Similarity Judgments
Source: ArXiv. 2024 Sep 5:arXiv:2310.07543v2. Originally published 2023 Oct 11. Preprint. [Version 2] (PMC10593068)
Supplement: Supplementary file 1 [file NIHPP2310.07543V2-supplement-1.pdf]

Supplementary Figure Legends

Supplementary Figure 1 (supplement to main text Figure 5)

Panel A. Behavior of I_{sym} for the 15-point configurations, a range of trials per triad (N , abscissa), and a range of decision rules (σ , standard deviation of added noise). Solid symbols indicate values of I_{sym} computed from simulated decisions. The dashed horizontal line indicates the *a priori* value for I_{sym} . Hollow boxes indicate values of I_{sym} computed from "flip any" surrogates, showing the ± 1 standard deviation range. The thin vertical lines are to aid visualization, and do not represent ranges. The beta-function prior (3.4) is used, with the value of the parameter a (shown in fine print over each data point) determined by maximum likelihood. Panel B expands the vertical scale.

Supplementary Figure 2 (supplement to main text Figure 6)

Panel A. Behavior of I_{umi} for the 15-point configurations, a range of trials per triad (N , abscissa), and a range of decision rules (σ , standard deviation of added noise). Solid symbols indicate values of I_{umi} computed from simulated decisions. The dashed horizontal line indicates the *a priori* value for I_{umi} . Hollow boxes indicate values of I_{umi} computed from surrogates, showing the ± 1 standard deviation range: wider boxes for "flip any" surrogates, narrower boxes for "flip all" surrogates. The thin vertical lines are to aid visualization, and do not represent ranges. The modified prior (3.27) is used, with the value of the parameter a (shown in fine print over each data point) determined by maximum likelihood and $h = 0.001$. Panel B expands the vertical scale.

Supplementary Figure 3 (supplement to main text Figure 7)

Panel A. Behavior of $I_{addtree}$ for the 15-point configurations, a range of trials per triad (N , abscissa), and a range of decision rules (σ , standard deviation of added noise). Solid symbols indicate values of $I_{addtree}$

computed from simulated decisions. The dashed horizontal line indicates the *a priori* value for $I_{addtree}$. Hollow boxes indicate values of $I_{addtree}$ computed from surrogates, showing the ± 1 standard deviation range: wider boxes for "flip any" surrogates, narrower boxes for "flip all" surrogates. The thin vertical lines are to aid visualization, and do not represent ranges. The beta-function prior (3.4) is used, with the value of the parameter a (shown in fine print over each data point) determined by maximum likelihood. Panel B expands the vertical scale.

Supplementary Figure 4 (supplement to main text Figure 8)

Behavior of $I_{addtree}$ for the 13-point configurations, a range of trials per triad (abscissa), and a range of decision rules (σ, standard deviation of added noise). Graphical conventions as in Supplementary Figure 4.

Supplementary Figure 5 (supplement to main text Figure 9)

Normalized values of I_{sym} (panel A), I_{umi} (panel B), and $I_{addtree}$ (panel C), for the 15-point configurations, a range of trials per triad (N, abscissa), and a range of decision rules (σ, standard deviation of added noise). Data from Supplementary Figures 1-4, replotted after normalizing the deviation from the *a priori* value by the number of trials per triad: $I_0 + (I - I_0)/N$, where I is the index from Supplementary Figures 1-4, I_0 is the *a priori* value, and N is the number of trials per triad. Graphical conventions as in Supplementary Figures 1-3.

Supplementary Figure 6 (supplement to main text Figure 10)

Dependence on the prior's shape parameter, a, for I_{sym} (panel A), I_{umi} (panel B), and $I_{addtree}$ (panel C), for the 15-point configurations and a range of trials per triad (N, abscissa). The decision rule $\sigma = 0.25$ is used. Column 1: a is determined by maximum likelihood; other columns: a is assigned the value

indicated over each column. The beta-function prior (3.4) is used for I_{sym} and $I_{addtree}$; the modified prior (3.27) is used for I_{umi} with $h = 0.001$. Other graphical conventions as in Supplementary Figures 1-3.

Supplementary Figure 7 (supplement to main text Figure 11).

Dependence on the prior's point mass parameter, h, for I_{sym} (panel A), I_{umi} (panel B), and $I_{addtree}$ (panel C), for the 15-point configurations and a range of trials per triad (N , abscissa). The decision rule $\sigma = 0.25$ is used. Column 1: h is determined by maximum likelihood (shown in fine print in lower row over each data point); other columns: h is assigned the value indicated over each column. The parameter a (shown in fine print over each data point) is determined by maximum likelihood in all cases. The modified prior (3.27) is used for all indices. Note that I_{umi} is undefined for $h = 0$ (panel B, second column). Other graphical conventions as in Supplementary Figures 1-3.

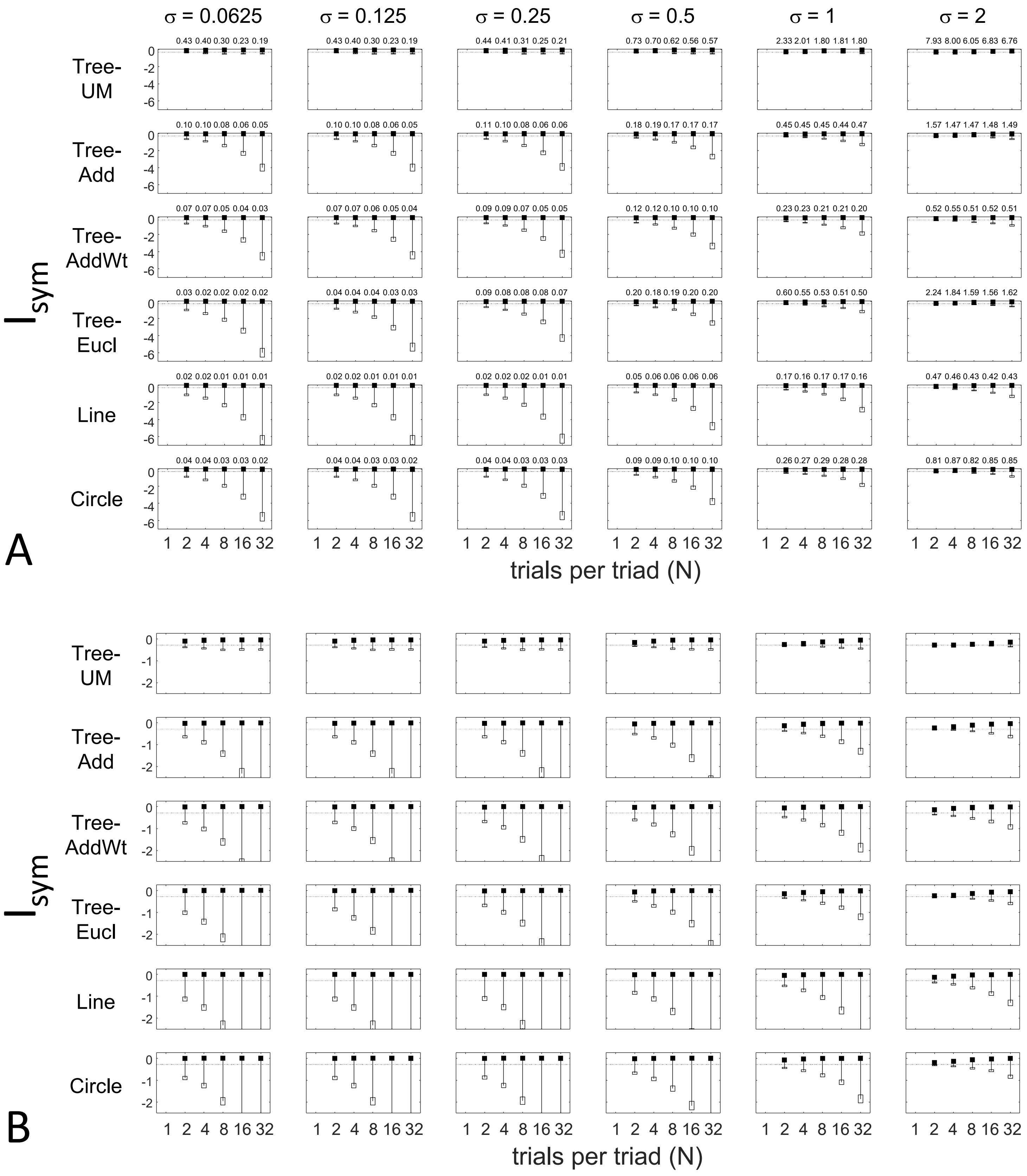

Supplementary Figure 1

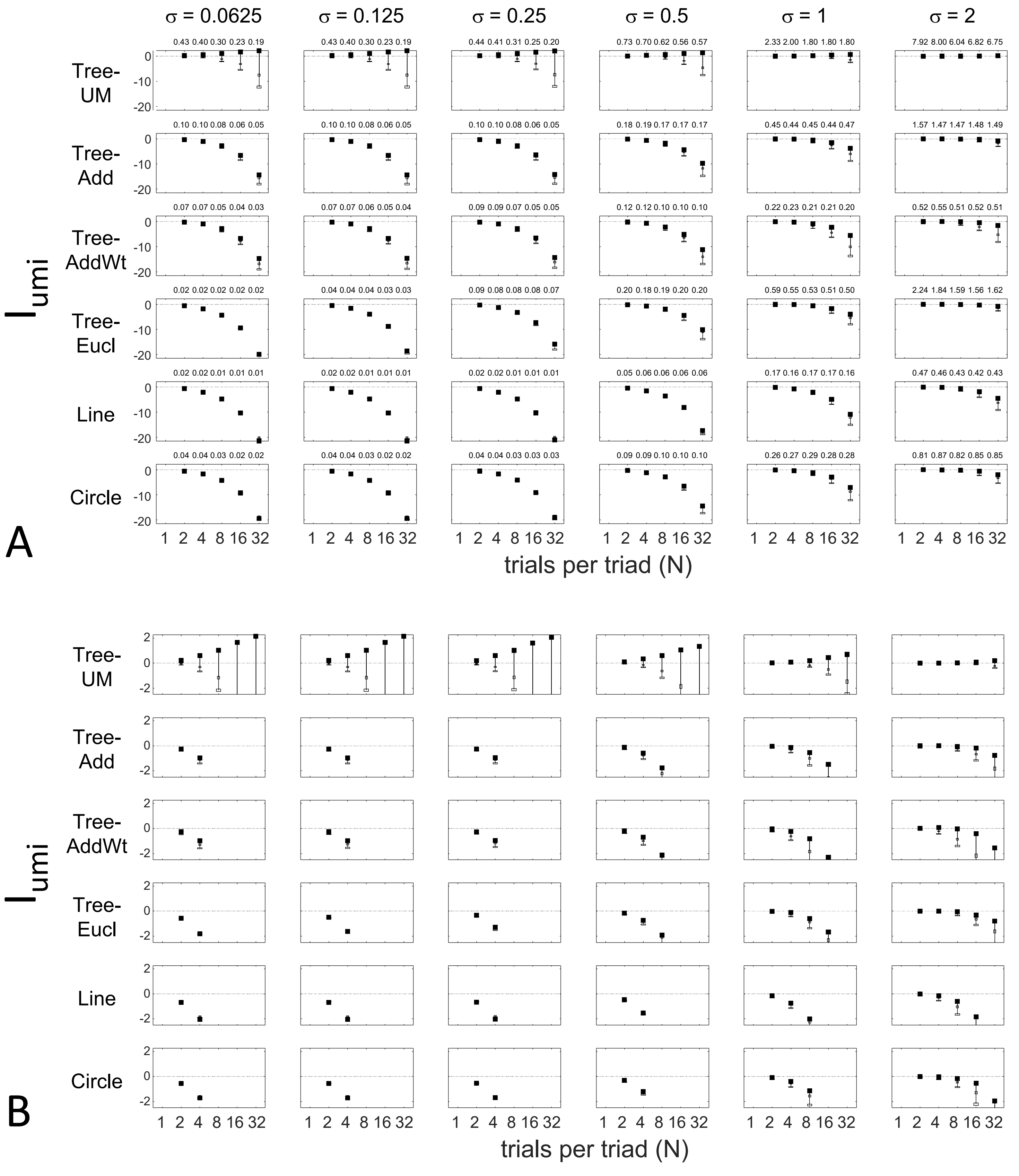

Supplementary Figure 2

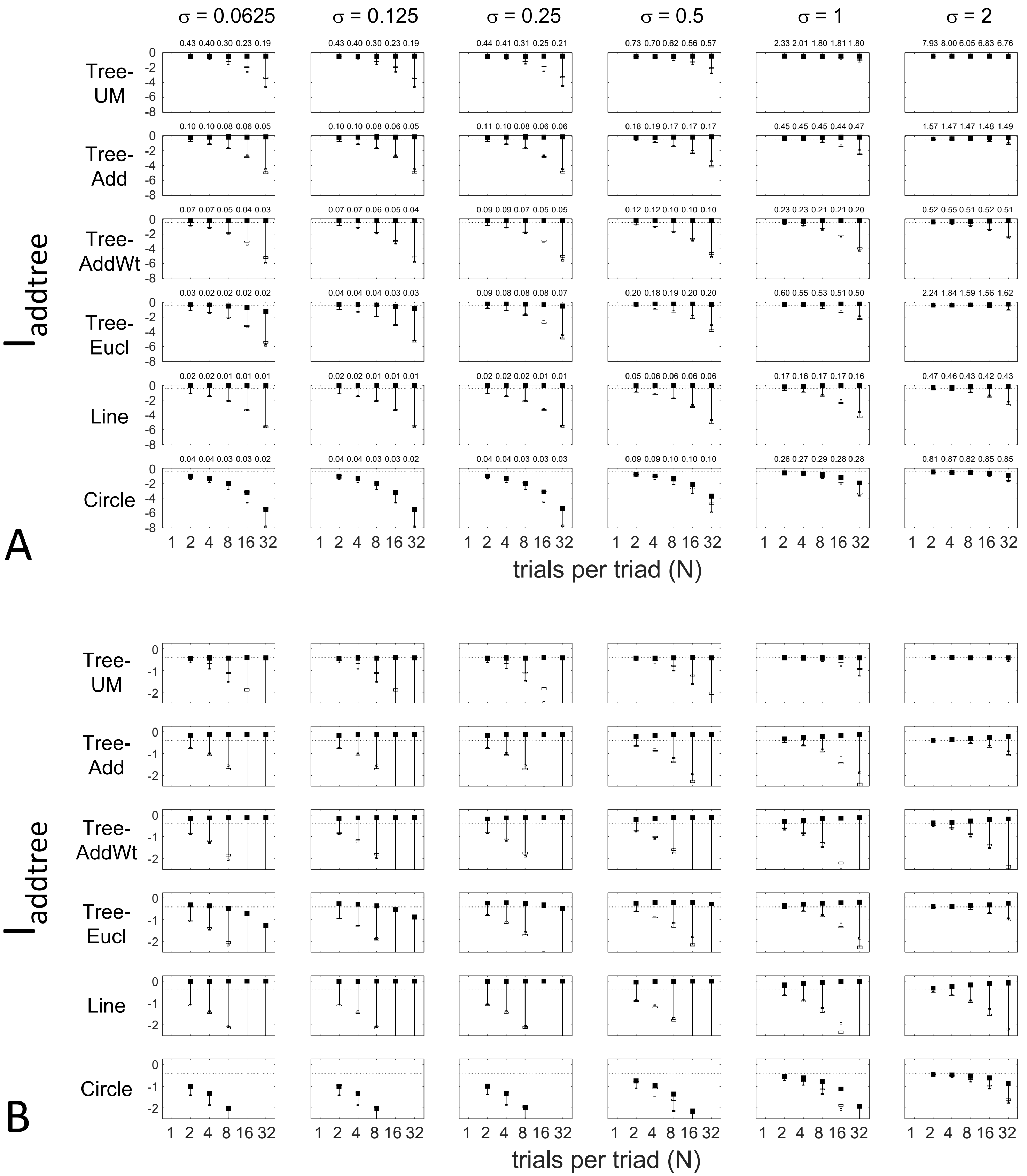

Supplementary Figure 3

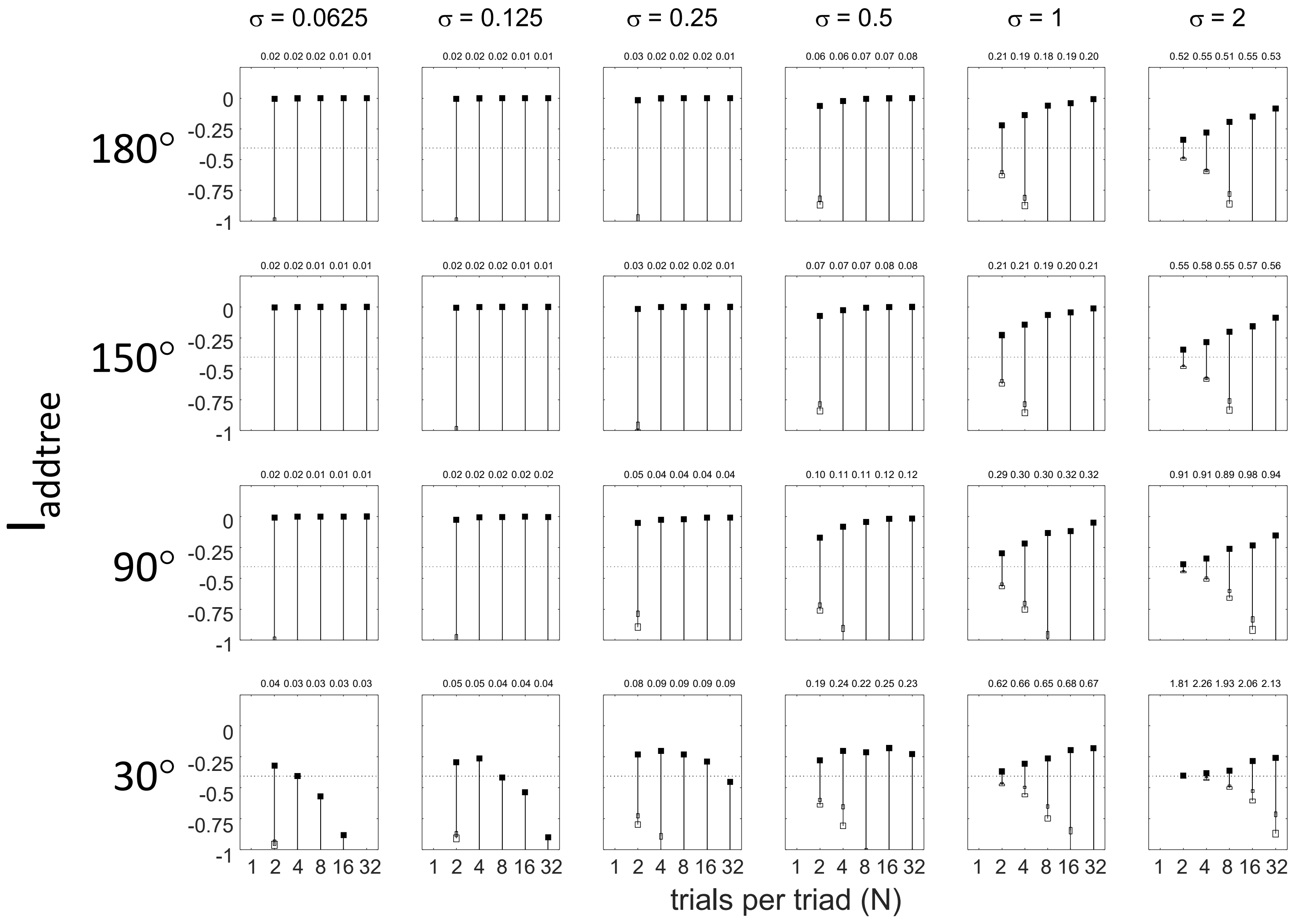

Supplementary Figure 4

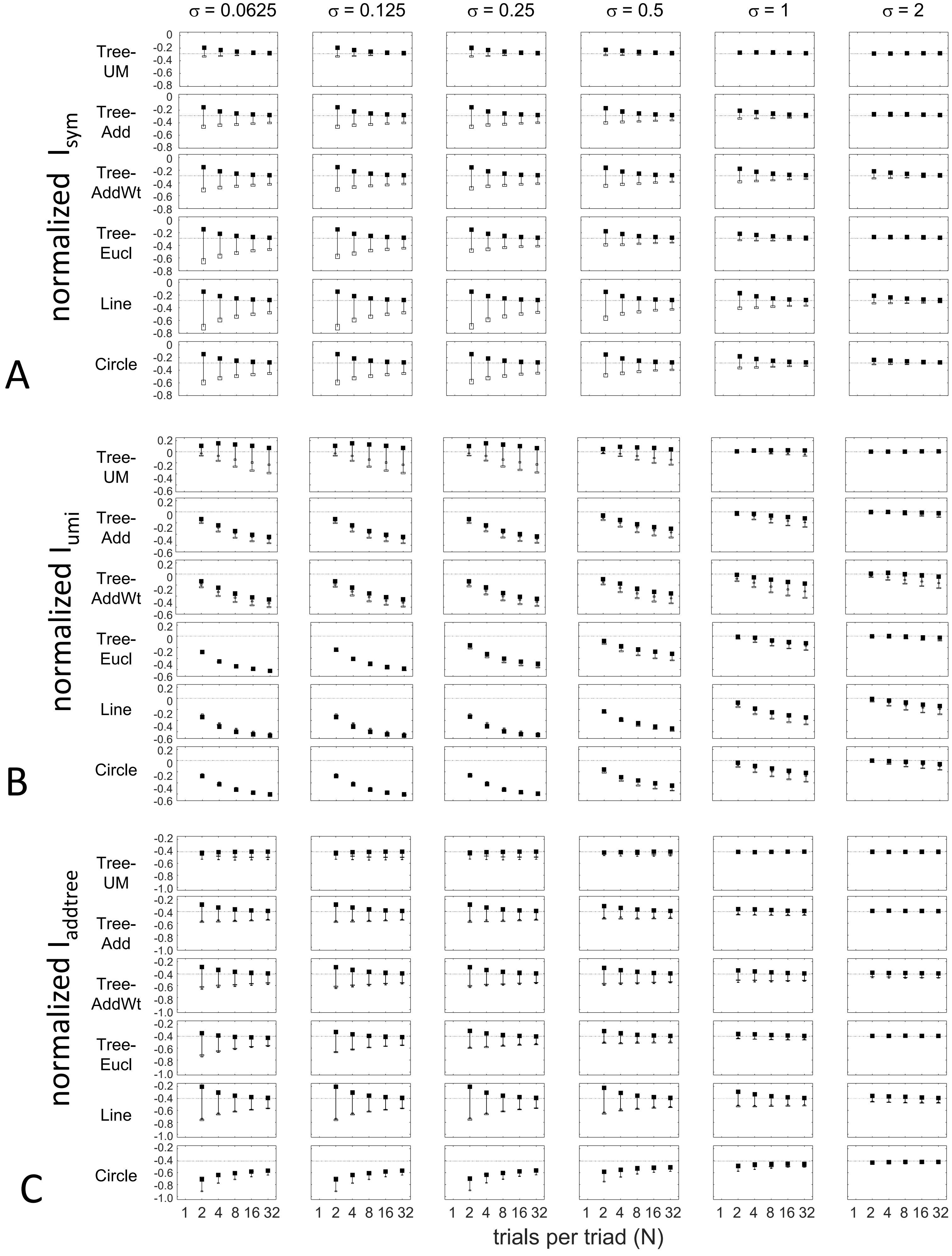

Supplementary Figure 5

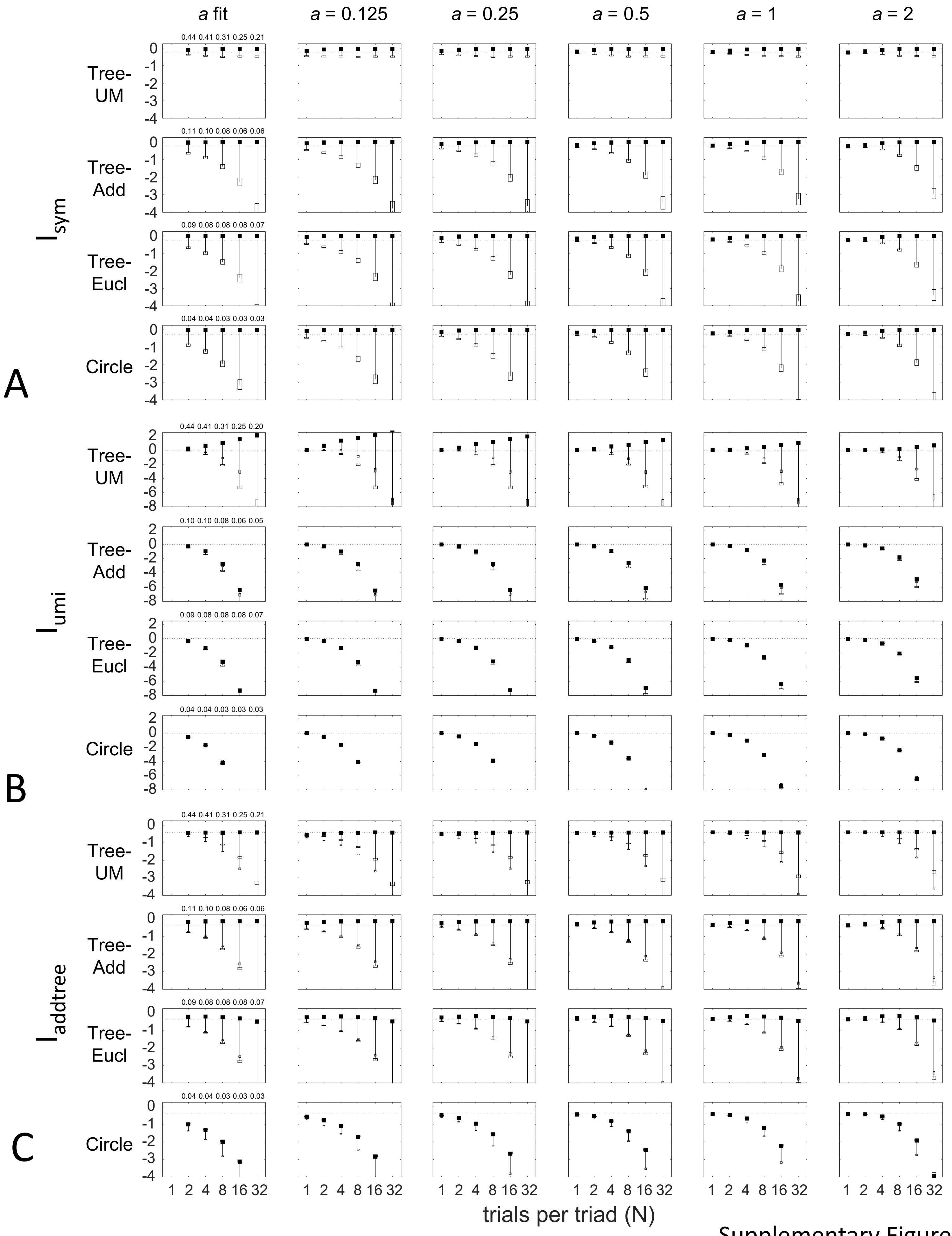

Supplementary Figure 6

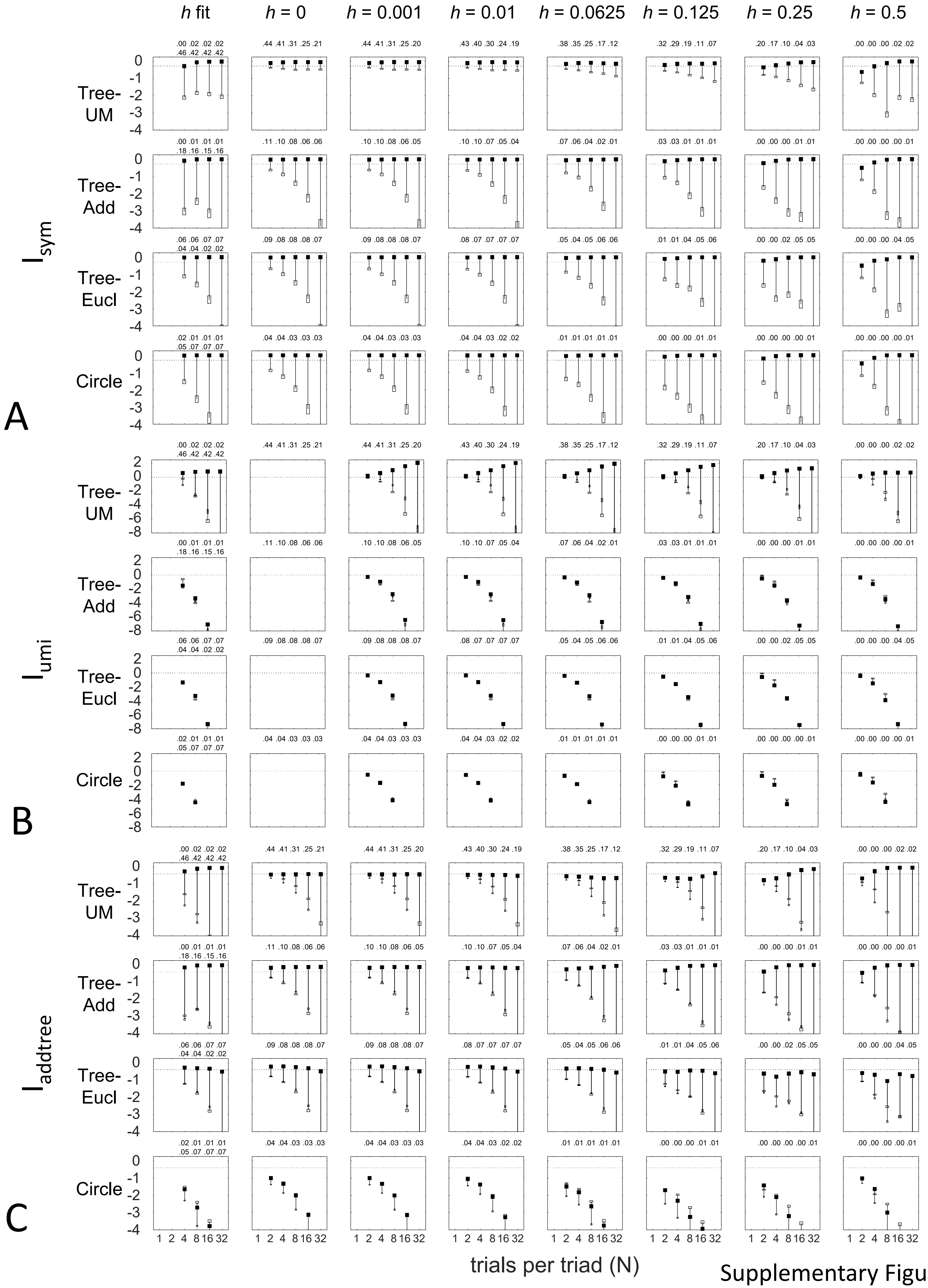

Supplementary Figure 7
